# Long‐Term Post‐Mitotic Culture of Human Fibroblasts Recapitulates and Accelerates Features of In Vivo Aging and Enables Dynamic Testing of Anti‐Aging Compounds

**DOI:** 10.1111/acel.70796

**Published:** 2026-11-01

**Authors:** Larissa Traxler, Lukas Karbacher, Oliver Borgogno, Taylor Ozbun, Kylie Champion, Anna Smaling, Lucia Zhou‐Yang, Joseph R. Herdy, Lena Böhnke, Barbara C. Böckle, Fred H. Gage, Hildegard I. D. Mack, Michaela Defrancesco, Jerome Mertens

**Affiliations:** ^1^ Department of Neurosciences University of California San Diego La Jolla California USA; ^2^ Sanford Consortium for Regenerative Medicine University of California San Diego La Jolla California USA; ^3^ Laboratory of Genetics The Salk Institute for Biological Studies La Jolla California USA; ^4^ Institute for Molecular Biology University of Innsbruck Innsbruck Austria; ^5^ Department of Dermatology, Venereology and Allergology Medical University of Innsbruck Innsbruck Tyrol Austria; ^6^ Department of Biochemistry and Genetics of Aging, Research Institute for Biomedical Aging Research University of Innsbruck Innsbruck Tyrol Austria; ^7^ Division of Psychiatry I, Department of Psychiatry and Psychotherapy Medical University of Innsbruck Innsbruck Tyrol Austria

## Abstract

Biological aging drives cellular dysfunction and human disease, yet studying human aging dynamics remains challenging due to limited experimental platforms. Here we show that long‐term post‐mitotic culture of human fibroblasts recapitulates and accelerates features of in vivo aging. Longitudinal paired transcriptomic and epigenetic analyses of four female fibroblast lines revealed that in vitro aging reflects key signatures of in vivo primary fibroblast aging. Direct neuronal conversion of in vitro‐aged fibroblasts preserved biological age, enabling pseudo‐longitudinal modeling of neuronal aging through direct reprogramming. Single‐cell transcriptomics of in vitro‐aging fibroblasts revealed a time‐dependent increase in age‐heterogeneity, consistent with the previously reported age‐associated heterogeneity driven by the variable loss of transcriptional programs in vivo. Using this accelerated aging platform, we evaluated anti‐aging compounds: Metformin broadly attenuated transcriptomic and epigenetic aging, while Rapamycin showed limited efficacy. These findings align with pre‐clinical evidence, providing a platform to evaluate and predict therapeutic anti‐aging compound efficacy at molecular resolution. This system advances our understanding of aging mechanisms and facilitates the development of interventions against age‐related diseases.

## Graphical abstract

Human fibroblasts undergo accelerated molecular aging in long‐term culture, recapitulating in‐vivo aging signatures and increasing age‐related heterogeneity. Direct neuronal conversion preserves biological age, enabling pseudo‐longitudinal studies and a scalable platform to evaluate anti‐aging interventions, including the differential effects of Metformin and Rapamycin.

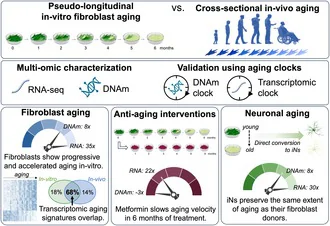

## 1 Introduction

Biological aging leads to decreased cellular functionality, an increase in illnesses and higher mortality risk. Due to unique human genetics, physiology, long lifespan, and human specificity of many aging‐related disorders, the predictive value of animal models is limited. Yet, models to dynamically study longitudinal human cellular aging are lacking. Human primary cell cultures and direct reprogramming technologies, such as directly induced neurons (iNs), enable the generation of donor‐ and patient‐specific cell models that closely resemble somatic cells and tissues (Rorteau et al. 2022; Mertens et al. 2015; Tang et al. 2017; Kabacik et al. 2022; Pitrez et al. 2024). However, while age‐equivalent models capture authentic human aging signatures of a donor, this cross‐sectional approach does not provide insights to the dynamic aging process or offer means to test intervention strategies against it. To trigger specific aging pathways in cell models, stressors such as chemical treatments including oxidative agents (Zhu et al. 2019), proteasome inhibition, senescence‐induction (Fathi et al. 2022; Nekrasov et al. 2016), or overexpression of aging‐related factors such as progerin (Miller et al. 2013; Vera et al. 2016) have been applied, but they are limited to the extent they can recapitulate the range and complexity of cellular aging. Meanwhile, the overall rate of aging strictly depends on the progression of time and thus the chronological age of an organism. Studying aging in long‐lived cells is particularly relevant for understanding how age‐associated changes accumulate in cell types that experience limited proliferative turnover, including post‐mitotic neurons and long‐lived dermal fibroblasts. Although fibroblasts retain proliferative capacity, they undergo relatively low rates of division and are exposed to replication‐independent stressors, including oxidative stress, mitochondrial dysfunction, inflammation, and epigenetic alterations, for decades. Thus, replicative senescence models, while valuable for understanding division‐associated aging processes, may not fully capture the mechanisms underlying physiological aging in long‐lived cell types (Tigges et al. 2014). Accordingly, our non‐replicative culture paradigm is intended to model a complementary, time‐dependent, and cell‐intrinsic component of fibroblast aging rather than to replace replicative senescence models. Understanding aging in non‐replicative cultures without clonal selection or replicative exhaustion/senescence is particularly relevant as they better resemble the natural in vivo aging situation.

In this study, we present a model system to induce aging in human primary fibroblasts in vitro by maintaining fibroblasts at confluency in a non‐replicative state for 6 months, inducing key transcriptomic and epigenetic signatures of in vivo aging without triggering replicative senescence. We observed substantial changes consistent with molecular aging including changes in DNA methylation (DNAm) and transcriptional aging clocks. Single‐cell transcriptomic analyses revealed an increasing heterogeneity in cellular aging over time, driven by the variable downregulation of specific transcriptional programs. This heterogeneity reflects in vivo aging dynamics and highlights the asynchronous nature of cellular aging within populations. Importantly, as fibroblasts are commonly the source of many direct trans‐differentiation protocols, which retain the age‐related signatures of the original cells (Mertens et al. 2015; Tang et al. 2017; Böhnke et al. 2019), this system facilitates pseudo‐longitudinal studies of age‐related diseases across various tissues. We demonstrate that pre‐aged fibroblasts can generate iNs that preserve their biological age, enabling modeling of neuronal aging. Using this platform, we further assessed the effects of two candidate anti‐aging compounds: (1) Metformin broadly attenuated transcriptomic and epigenetic aging, while (2) Rapamycin showed limited effects. These results align with existing pre‐clinical findings, indicating a utility of this platform to evaluate candidate age‐modulating compounds (Yang et al. 2024; Neff et al. 2013). Together, our work establishes a robust framework for studying human aging mechanisms and developing interventions for age‐related diseases.

## 2 Results

### 2.1 Long‐Term In Vitro Culture to Recapitulate Features of In Vivo Fibroblast Aging

Here, we set out to explore a long‐term non‐dividing in vitro culture as a model for cellular aging that allows for a dynamic investigation of the aging process by mapping and directly comparing in vivo aging with in vitro aging signatures within the same cell type using genome‐wide DNAm and RNA sequencing (RNA‐seq) analyses (Figure 1a). We first obtained primary human fibroblasts from skin punch biopsies from 33 individuals aged 0 to 92 years (Supplementary Figure 1a). To control for changes that are due to the in vitro culture environment, the 33 in vivo‐aged cell lines were harvested before reaching passage 10 and assessed at full confluency. For the in vitro aging experiments, fibroblasts from four independent female donors aged 53, 55, 67, and 68 years were cultured at confluency without passaging for 6 months (Supplementary Figure 1b). This age range corresponds to the period during which age‐related diseases most commonly begin to manifest in humans and is virtually inaccessible with conventional aging models. Long‐term cultures of confluent contact‐inhibited non‐dividing fibroblasts remained stable without apparent morphological changes, and did not show signs of overgrowth or cell death (Figure 1b). In contrast to sequential passaging experiments (Kabacik et al. 2022), fibroblasts maintained in long‐term culture do not enter a senescence‐associated state, confirmed by the absence of p21 and p16 immunoreactivity, maintenance of small nuclear sizes, and no upregulation of genes associated with senescence and the senescence‐associated secretory phenotype (SASP) (Figure 1b,c; Supplementary Figure 1c–e). In addition, fibroblasts retained proliferative capacity and remained passagable after 6 months of in vitro aging, demonstrating that long‐term culture did not induce permanent cell cycle arrest. To quantify biological aging signatures, we first employed DNAm and transcriptomic clock analyses of our in vivo‐ and in vitro‐aged samples. First, we compared different established DNAm clocks: The DNAm Blood&Skin clock (Horvath 2013) outperformed the Hannum clock (Hannum et al. 2013), the PhenoAge clock (Levine et al. 2018), and the Horvath multi‐tissue DNAm clock in cultured human fibroblasts, showing a near‐perfect correlation of DNAm age to the chronological age of the 22 in vivo‐aged donor samples (*R* ^2^ = 0.98, *p* = 7.2^−15^; Supplementary Figure 1f,g). The Blood&Skin clock only minimally overestimated chronological age with a velocity of 1.01×, while the other tested clocks underestimate chronological age, and showed lower aging velocities (Hannum: 0.40×, PhenoAge: 0.48×; Supplementary Figure 1f,g). The aging velocity (linear regression slope) reflects the rate of molecular aging per year, with steeper slopes indicating faster aging and flatter slopes indicating slower progression. Given its accuracy, we continued to use the Blood&Skin DNAm clock to assess DNAm ages in this study. However, while remarkably accurate, epigenetic clocks utilize DNAm sites in the genome that are rarely associated with genes, rendering their biological interpretation difficult (Malousi et al. 2018). By contrast, transcriptomic signatures are more directly related to cellular functions, and meta‐analysis of transcriptional aging data revealed that chronological age can also be predicted based on RNA‐Seq data (Ren and Kuan 2020). We thus tested five previously established transcriptomic clocks in our in vivo cohort (*n* = 20, 22–92 years), and in the larger fibroblast cohort of Fleischer et al. (*n* = 143, ages 1–96 years) (Fleischer et al. 2018). We first probed for transcriptomic age using the RNAAgeCalc (Ren and Kuan 2020), of which one version is trained on an extensive dataset of 26 different tissues (RNAAgeCalc—across‐tissue), while the other is trained on fibroblast data (RNAAgeCalc—fibroblast‐specific). Both transcriptomic clocks utilize genes that participate in hallmark processes of aging, such as proteasome and ribosomes, mitochondrial and metabolic pathways, inflammation, and DNA replication and repair (Ren and Kuan 2020; Peters et al. 2015). While the across‐tissue and fibroblast‐specific clocks both ticked slower than chronological time, with an aging velocity of 0.24× (across‐tissue) and 0.19× (fibroblast‐specific), the across‐tissue clock was more accurate (*R* ^2^ = 0.21, *p* ≤ 0.0001) than the fibroblast‐specific version (*R* ^2^ = 0.19, *p* ≤ 0.0001), especially in the smaller in vivo cohort (no‐tissue *p* = 0.08, fibroblast *p* = 0.61) (Supplementary Figure 1h,i). We additionally tested an independent multi‐tissue transcriptomic age estimator (tAge) (Tyshkovskiy et al. 2026). The tAge clock outperformed the RNAAgeCalc (*R* ^2^ = 0.57, *p* < 0.0001) and showed significant correlation with chronological age in both in vivo datasets (Supplementary Figure 1j). In contrast, the transcriptomic clock trained specifically on blood, developed by Peters et al., showed weaker associations with chronological age in fibroblasts (Supplementary Figure 1k) (Peters et al. 2015). This suggests that the multi‐tissue transcriptomic clocks, including tAge and the RNAAgeCalc—across‐tissue (thereafter simply called RNAAgeCalc) are useful for predicting age in cultured fibroblasts from donors of different ages. We therefore focused on these two transcriptomic clocks for our study to assess the influence of long‐term non‐dividing in vitro culture on aging signatures in primary human fibroblasts.

**FIGURE 1:**
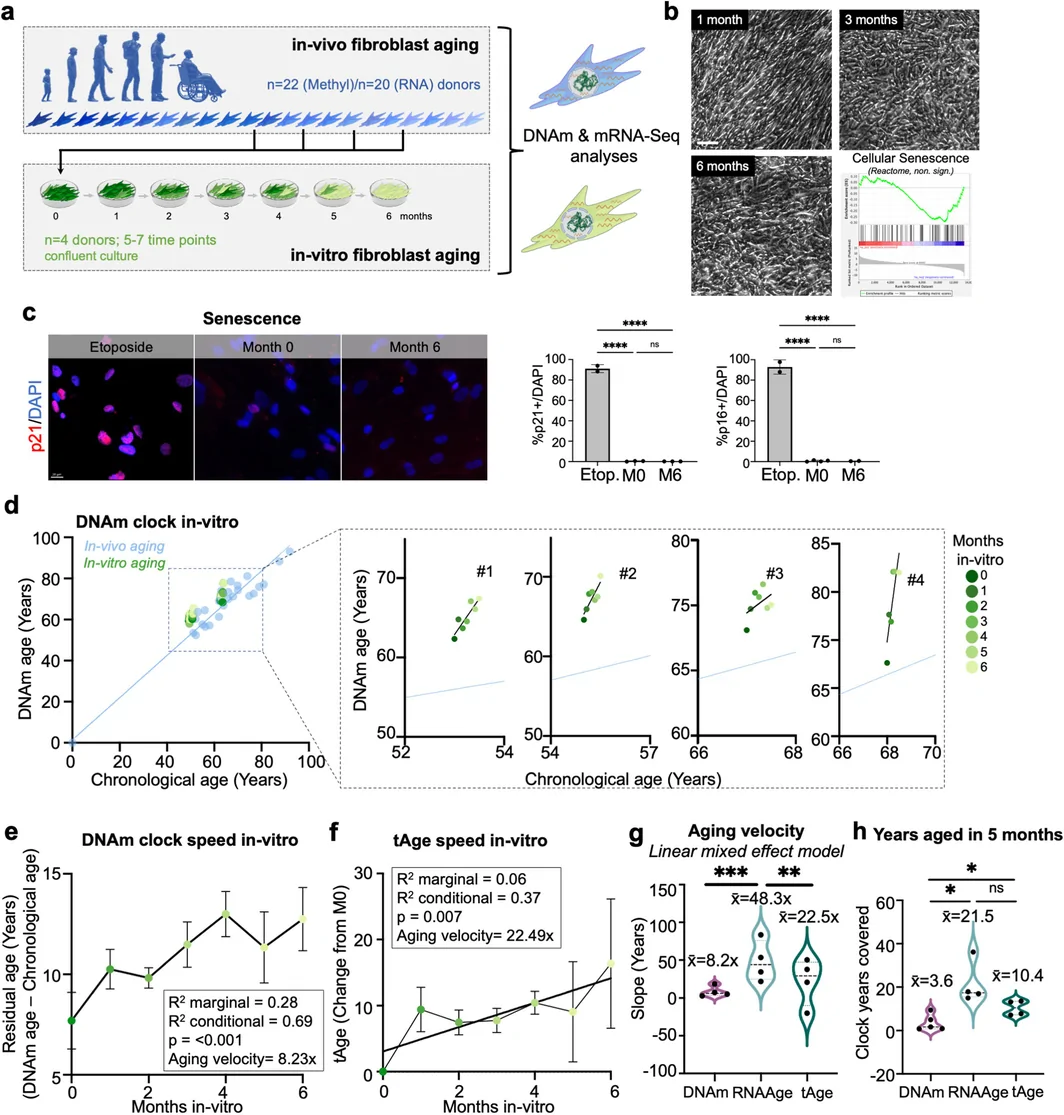
DNAm and transcriptomic clocks comparing in vivo and in vitro aging. (a) Human fibroblasts of donors aged 0–92 years were harvested once reaching confluency (in vivo aging). Four independent donors aged 53, 55, 67, and 68 were grown to confluency (time‐point 0) and kept in culture without passaging for 6 months. RNA‐seq and DNAm EPIC Array were performed at 5–7 time‐points each 30 days. (b) Phase contrast images taken at time of harvest each month. Gene set enrichment analysis related to Reactome pathway “Cellular Senescence” in in vitro aged cells (NES = 0.98, FDR adjusted *p*‐value = 0.49). (c) Immunofluorescence staining (p21) of fibroblasts at Months 0 and 6. Positive control represents senescent fibroblasts (Etoposide, 20 μM, 48 h). Percentage represents p21+ and p16+ nuclei/total number of nuclei (DAPI). Ordinary One‐way ANOVA and Tukey's multiple comparisons test (p21: *F*(2/5) = 133.6, *p* < 0.0001; p16: *F*(3/7) = 647.6, *p* < 0.0001). (d) Blood&Skin DNAm age applied to methylation data of in vitro aging (*n* = 4 independent samples). Blue dots represent in vivo aged donors, with the blue line indicating the corresponding in vivo linear regression. In vitro aged donors are shown in green (dark green for M0 and light green for M6), with the black lines representing the linear regression per donor (mean slope in vivo = 1.01, mean slope in vitro = 8.23, 95% CI [3.9, 15.1]). (e) DNAm age of in vitro aging, standard deviation represents variance between donors. Residual age was calculated by subtracting the chronological age from the estimated DNAm age. Linear mixed effects model of in vitro aging with donor as random variable. (f) Transcriptomic tAge based on in vitro aging. Age was calculated by subtracting Month 0 from each timepoint per donor. Linear mixed effects model of in vitro aging with donor as random variable. (g) Aging velocity, defined as the slope of the linear regression in years, was calculated for DNAm clock, RNAAgeCalc—across‐tissue (RNAAge), and tAge for in vitro aging. Each dot represents an independent donor. Differences in slopes were assessed using linear mixed effect model with donor as random variable. (h) Difference in DNAm (*n* = 4 independent donors, one technical replicate for Donor #2) and transcriptomic age (*n* = 4 independent donors) of Month 5 compared to baseline (M0). Mixed‐effects model (REML) with individuals as random effects, *F*(0.9663, 2.899) = 16.05. Tukey's multiple comparison test. Each dot represents an individual sample. Significance: *p < 0.05, **p < 0.01, ***p < 0.001, ****p < 0.0001.

### 2.2 In Vitro Aging Accelerates DNAm and RNA Aging Clock Velocities

Next, we extracted DNA and RNA each month from our in vitro aging cohort and applied the clock‐based age prediction (*n* = 4 donors; 5–7 time points per donor) (Figure 1a). The donors began the experiment with different levels of DNAm age acceleration, ranging from 4.6 and 6.0 DNAm years in the lower‐passage Donors #3 and #4, to 9.6 and 10.7 DNAm years in the higher‐passage Donors #1 and #2 (Figure 1d). This variation may likely reflect differences in time in culture before the formal start of the experiment (Supplementary Figure 1b). During extended post‐mitotic in vitro culture, the DNAm clock continued to tick, but at an accelerated pace in vitro for all donors (Figure 1d). To quantify the aging velocity of DNAm age estimates across time in vitro, we employed linear mixed effect models with donor as a random effect and found that DNAm age increases significantly with time in vitro, revealing an average aging velocity of 8.2× (Figure 1e; Supplementary Figure 1m) and a significant increase in DNAm age after 6 months in culture compared to Month 0 (Supplementary Figure 1n). Also the transcriptomic clock showed an increase in aging as a function of in vitro culture time, with an aging velocity of 22.5× using tAge, and 48.3× based on the RNAAgeCalc (Figure 1f,g, Supplementary Figure 1o), but with substantially higher variability across donors. Prolonged in vitro cultures significantly increased age across transcriptomic clocks (Supplementary Figure 1p). Interestingly, aging velocities significantly differ between DNAm and transcriptomic clocks, indicating that the transcriptomic clock progresses ~3–5‐fold faster than the DNAm clock (Figure 1g). A paired comparison of predicted age difference over 5 months in vitro revealed a significant difference in epigenetic and transcriptomic age gained, with a gain of 3.6 epigenetic years (95% CI [−1.0, 8.2]) and 21.5/10.4 transcriptomic years (95% CI [5.8, 37.2]/[4.9, 15.9]) (Figure 1h). We next assessed changes in relative transcriptional age using the RNAge clock, which was designed to detect changes in the relative transcriptomic age of human cells (Zhang et al. 2025). Comparing young and old donors in our cohort and the in vivo cohort from Fleischer et al., we observed a marked increase in RNAge, particularly for the pan and primary fibroblast markers (Supplementary Figure 1l). In vitro aging similarly resulted in a strong increase in RNAge from Month 0 to 6, demonstrating that the increase in transcriptomic age was consistently captured across age estimators (Supplementary Figure 1l). These data demonstrate that key epigenetic and transcriptomic features of in vivo aging are recapitulated and accelerated in vitro, and that global transcriptomic aging accelerates to a higher velocity than the epigenetic aging process.

### 2.3 In Vitro Aging Recapitulates Key Features of In Vivo Transcriptomic Aging

We asked to what extent in vitro aging reflects in vivo signatures of cellular aging on the transcriptional pathway level. To assess the transcriptional overlap of biological processes associated with aging in vivo and in vitro, we performed linear regression analyses on RNA‐seq data from in vitro aging, in vivo aging (*n* = 20, ages 22–92 years, adjusted for sex), and the larger in vivo cohort by Fleischer et al. (GSE113957; *n* = 143 donors, ages 1–96 years, adjusted for sex) (Fleischer et al. 2018) (Figure 2a). To identify aging genes, multiple‐testing correction was applied for both datasets, whereas unadjusted *p*‐values were used to define aging associated genes in the smaller in vivo cohort to retain potential biological signals despite limited statistical power (Supplementary Figure 2a). Gene‐level gene‐set enrichment analysis revealed strong enrichment of up‐ and downregulated aging genes in both in vivo datasets, indicating that despite limited power, the smaller in vivo cohort captures relevant aging signatures (Supplementary Figure 2b). Consistently, in vitro aging showed significant enrichment with gene sets derived from both in vivo cohorts (Supplementary Figure 2b). We next sought to identify overlapping aging pathways. Significant aging genes of the three aging cohorts were assessed simultaneously using Cytoscape (ClueGO) (Figure 2b). This strategy leverages the statistical power of the larger dataset while preserving the biological relevance of the in vivo samples processed using the same experimental pipeline as the in vitro aging fibroblasts. KEGG pathway enrichment was conducted to compare age‐associated genes from in vitro and in vivo aging. Using ClueGO, we quantified the relative contribution of up‐ and downregulated gene lists from each dataset to the enriched KEGG pathways, where 33% indicates equal contribution from all three lists, and 0% or 100% indicates that all pathway genes originate from a single list (Supplementary Figure 2c,d). To confirm that the overlapping KEGG pathways were significantly enriched within each dataset, we performed gene‐set enrichment analysis for each pathway identified by ClueGO. Pathways with at least 15% contribution from each dataset were considered overlapping. This analysis revealed 17 overlapping downregulated aging pathways shared across all three datasets (Fisher's exact test; in vitro vs. in vivo: *p* = 1.25e^−13^, odds ratio = 22.2, 95% CI [8.9, 58.4]; in vitro vs. Fleischer: *p* = 2.2e^−16^, odds ratio = 29.3, 95% CI [12.5, 74.2]; Fleischer vs. in vivo: *p* = 2.2e^−16^, odds ratio = 37.7, 95% CI [13.7, 122.1]), as well as seven overlapping upregulated aging pathways (Fisher's exact test; in vitro vs. in vivo: *p* = 1.67e^−05^, odds ratio = 16.0, 95% CI [4.3, 60.6]; in vitro vs. Fleischer: *p* = 1.88e^−12^, odds ratio = 52.6, 95% CI [15.4, 204.8]; Fleischer vs. in vivo: *p* = 2.2e^−16^, odds ratio = 550.6, 95% CI [55.5, 16384.0]) (Figure 2b). Genes contributing to the overlapping KEGG pathways identified by ClueGO were defined as longitudinal aging (LTA) genes (Supplementary Tables 1 and 2). Of the pathways significantly associated with in vitro aging, 60 of the 66 significant downregulated aging pathways, and 20 of the 31 significant upregulated pathways were also significantly associated with in vivo aging. The remaining pathways likely reflect culture‐specific and donor‐specific transcriptional responses, thereby defining the scope and limitations of this reductionist model. Thus, long‐term non‐replicative culture recapitulates a substantial, reproducible subset of conserved aging‐associated programs rather than the full complexity of in vivo fibroblast aging.

**FIGURE 2:**
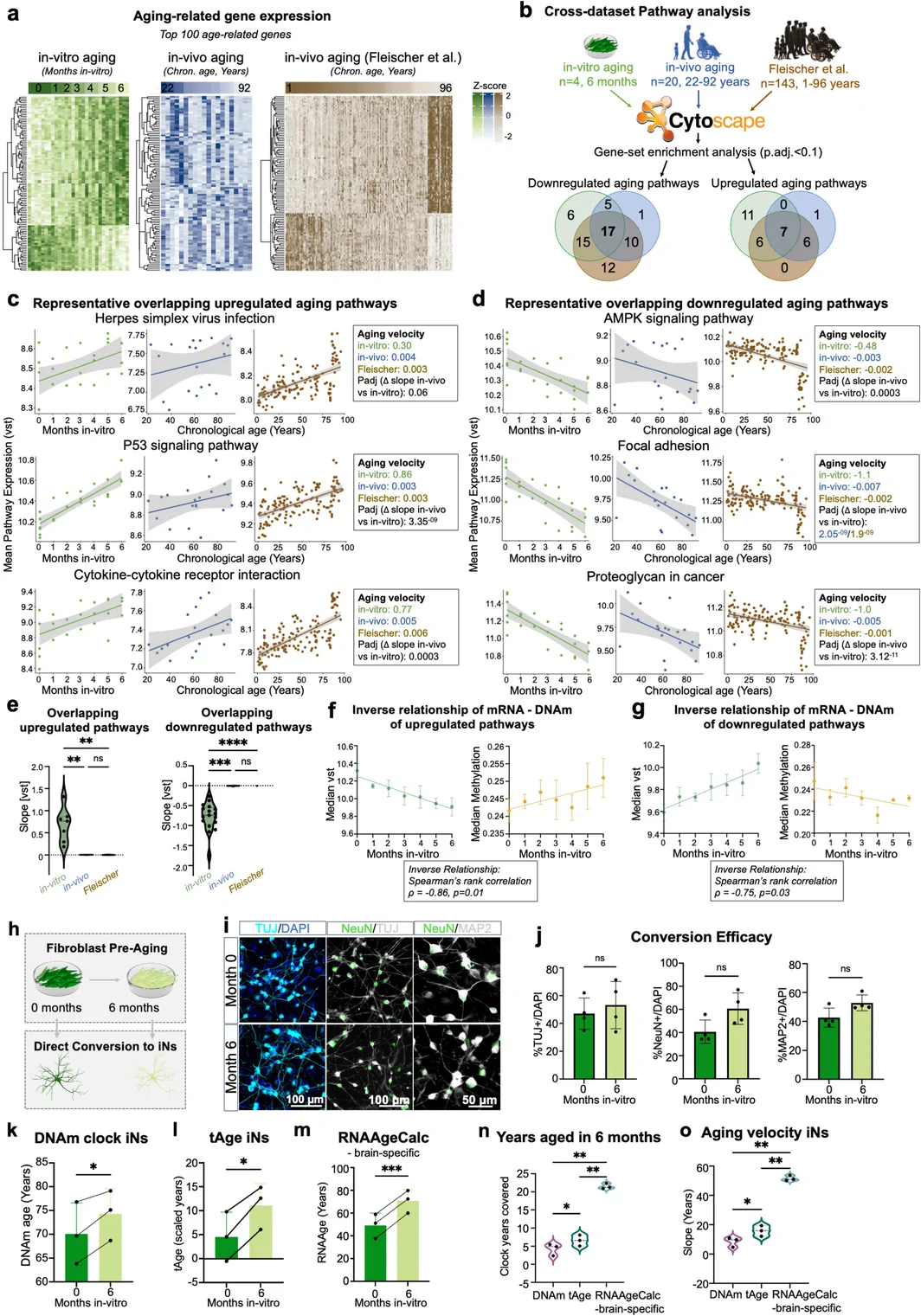
In vitro aging transcriptome changes are concordant with human fibroblast aging in vivo. (a) Linear regression of gene expression with age. In vivo aging analyses were adjusted for sex (model: Age + sex). The top 100 up‐ and downregulated genes along in vitro aging (green), in vivo aging (blue), and Fleischer et al. in vivo aging (brown) are shown. (b) Schematic of overlapping pathway analysis performed using CLUEGO (Cytoscape). Up‐ and downregulated aging genes were analyzed separately for in vitro aging (adjusted *p* < 0.05), Fleischer et al. in vivo aging (adjusted *p* < 0.05), and our in vivo aging dataset (*p* < 0.05). Gene‐set enrichment analysis was used to determine significance (adjusted *p* < 0.1; fgsea, R). Venn diagram depicts overlap among significantly enriched pathways. (c, d) Representative accelerated up‐ and downregulated KEGG pathways. Vst‐normalized expression was averaged per pathway, and linear regression was used to estimate pathway‐level slopes. To assess pathway‐level differences between in vitro and in vivo aging at gene‐level, we fit a linear model including an interaction term between age and dataset (Age × Dataset) with in vitro aging as reference. Interaction *p*‐value was corrected for multiple testing using the Benjamini–Hochberg method. (e) Average slopes of genes contributing to upregulated (*n* = 7) and downregulated (*n* = 17) pathways. Each dot representing one pathway; slopes were calculated based on changes in vst‐normalized gene expression per year. For upregulated pathways, data met normality assumptions and were analyzed using Brown‐Forsythe ANOVA (df = 2, *p* = 0.0017) followed by Dunnett's multiple comparisons test (in vitro vs. in vivo *p* = 0.009, in vitro vs. Fleischer *p* < 0.009 in vivo vs. Fleischer *p* = 0.97). For downregulated pathways, normality assumptions were not met and differences were assessed using Kruskal‐Wallis test (*H* = 37.71, df = 2, in vitro vs. in vivo *p* = 0.0002, in vitro vs. Fleischer *p* < 0.0001, in vivo vs. Fleischer *p* = 0.11). (f, g) Gene expression (green) and corresponding promoter methylation (orange)—defined as methylation at TSS200, TSS1500, 5′UTR, and 1st exon regions—of genes contributing to upregulated (*n* = 7) and downregulated (*n* = 17) aging pathways in in vitro aging. Values were averaged per pathway. Standard deviation represents variance between pathways. (h) iN‐competent fibroblasts (stable UNA expression) were pre‐aged with the established protocol and converted to iNs at baseline (M0) and after 6 months of in vitro aging. (i) Immunofluorescence staining of iN cultures for neuronal markers after 21 days of conversion induction. (j) Conversion efficacy at Day 21 of conversion. Each dot represents an independent donor, based on the average of 2–3 images per donor. TUJ: Paired *t*‐test (*t*(3) = 0.74, 95% CI [−20, 32]). MAP2 and NeuN did not meet normality assumption, Wilcoxon test was performed on 4 paired samples (NeuN *W* = 10, median difference = 13; MAP2 *W* = 10, median difference = 9). (k–m) Epigenetic and transcriptomic age calculations (*n* = 3 per time‐point). (k) Blood&Skin DNAm clock. Each dot represents an individual donor. Paired *t*‐test (*t*(2) = 4.4, 95% CI [0.1, 8.3]). (l) tAge. Paired *t*‐test (*t*(2) = 7.6, 95% CI [2.8, 10.3]). (m) RNAAgeCalculator—brain‐specific. Paired *t*‐test (*t*(2) = 49.7, 95% CI [19.6, 23.4]). (n) Subtraction of baseline estimated age from age at month six. Repeated measures ANOVA, *F*(1, 21, 2.24) = 434.5, *p* = 0.0013, Tukey's multiple comparisons test. (o) Aging velocity as calculated by simple linear regression per donor (slope). Repeated measures ANOVA, *F*(1, 25, 2.5) = 565.7, *p* = 0.0005, Tukey's multiple comparisons test. (e, j–o) Significance: *p < 0.05, **p < 0.01, ***p < 0.001, ****p < 0.0001.

We focused subsequent analyses on the overlapping aging‐associated pathways, representing conserved transcriptional programs consistently altered across both in vitro and in vivo aging datasets. Accordingly, pathway‐level aging velocities reflect the shared component of aging‐associated transcriptional change, thereby avoiding confounding by cohort‐ or model‐specific aging signatures. Variance‐stabilizing transformation (vst)‐normalized counts were averaged per pathway prior to pathway‐level linear regression modeling. Consistent with the observed acceleration based on transcriptomic aging clocks, the majority of the overlapping pathways were accelerated several‐fold in vitro (Figure 2c,d). Wilcoxon rank‐sum tests comparing gene‐level slopes within each pathway revealed that all pathways were significantly accelerated in vitro (adjusted *p* < 0.1; Figure 2c–e, Supplementary Figure 2e–g). In contrast, both in vivo aging datasets showed comparable aging velocities across all pathways (Figure 2e). Together, these results indicate that our in vitro model accelerates transcriptional aging programs without distorting the direction of concordant aging pathways identified across datasets, consistent with the global transcriptomic trend of in vitro aging, which displayed significantly greater slope changes compared to in vivo aging (Supplementary Figure 2h).

Next, to integrate potential promoter methylation (DNAm) changes in aging gene promoters with mRNA expression, we first selected genes associated with all 24 overlapping pathways of in vitro and in vivo aging and calculated their promoter methylation based on the average methylation of TSS1500, TSS200, 5′UTR and 1st exon (Ren and Kuan 2019). Spearman's rank correlation test revealed a strong inverse relationship between RNA expression and DNA methylation for genes contributing to the downregulated (*ρ* = −0.86, *p* = 0.01), and upregulated pathways (*ρ* = −0.75, *p* = 0.03) (Figure 2f,g).

Together, these data show that in vitro aging recapitulates and accelerates key in vivo transcriptome signatures, and that transcriptomic aging velocities substantially vary across different pathways. The data further emphasize the notion that while in vitro transcriptomic aging velocities seem to uncouple from DNAm in terms of velocity, they remain functionally coupled.

### 2.4 Sex‐ and Age‐Stratification Have Minimal Impact on Aging Transcriptome Signatures

To evaluate the potential impact of sex on the aging‐associated transcriptome signatures, we performed linear regression analysis with and without adjustment for sex. The overlap between sex‐adjusted and non‐adjusted results was highly significant (in vivo aging: *p*‐value < 2.2e^−16^, odds ratio = 585, 95% CI [460, 750]; GSE113957: *p*‐value < 2.2e^−16^, odds ratio = 2698, 95% CI [2100, 4300]), indicating that sex had no major effect on the identified aging signatures. Likewise, stratifying donors into young (< 40 years, *n* = 67) and old (> 40 years, *n* = 76) groups revealed no difference in aging velocity between the two groups, showing a strong concordance between them (*R* ^2^ = 0.876, *p* = 0; robust Pearson correlation, removal of 1.5× IQR, Supplementary Figure 2i).

### 2.5 Direct Neuronal Conversion of In Vitro‐Aged Fibroblasts Generates a Pseudo‐Longitudinal iN Model

Direct conversion of fibroblasts from aging human donors into age‐equivalent iNs has been instrumental in studying age‐related neuronal phenotypes (Mertens et al. 2021; Traxler et al. 2022; Herdy et al. 2022; Victor et al. 2018; Sun et al. 2024). However, obtaining true longitudinal samples from the same donors over extended periods to capture aging and disease trajectories remains challenging. To test whether in vitro aged fibroblasts could serve as a starting source for iNs with increased aging signatures, we cultured fibroblasts from three donors for 6 months before converting them into iNs using our established protocol (Zhou‐Yang et al. 2021) (Figure 2h). To first assess whether time in vitro affects neuronal conversion efficacy, we converted fibroblasts from Month 0 and 6 into iNs and stained the cultures with the neuronal markers β‐III‐tubulin (TUJ), NeuN, and MAP2 (Figure 2i). We observed no significant differences in neuronal conversion efficiency between the two time points (Figure 2j), which is consistent with the absence of a senescence phenotype because senescent fibroblasts do not convert into neurons efficiently (Wang et al. 2025; Jiang et al. 2015).

We next assessed whether aging‐associated molecular signatures acquired during in vitro culture were retained following neuronal conversion. DNAm and transcriptomic clock analyses revealed a clear increase in biological age in iNs generated from 6 months pre‐aged fibroblasts compared to iNs generated from baseline fibroblasts (Figure 2k–o, Supplementary Figure 2j). DNAm age increased by an average of 4.2 ± 1.7 years (Figure 2k), while the tAge and RNAAgeCalc increased by 6.6 ± 1.5 and 10.7 ± 4.4 years, respectively (Figure 2l, Supplementary Figure 2j). To determine whether this accelerated aging signature was maintained following replating of the pre‐aged fibroblasts, we repeated neuronal conversion after transferring the fibroblasts to fresh culture dishes at Month 6. The accelerated aging signature was preserved to a similar extent following replating, indicating that the age‐related molecular changes acquired during pre‐aging were robustly retained (Supplementary Figure 2k).

To assess the performance of the clocks across the parental fibroblasts and resulting iNs, we additionally compared age acceleration between the two cell types. DNAm age showed largely consistent changes, with an average gain of 5.7 years in fibroblasts and 4.2 years in iNs over 6 months of in vitro aging. Transcriptomic clocks showed a greater increase in fibroblasts than in iNs, with tAge increasing by 17.7 versus 6.6 years (*p* = 0.5) and the RNAAgeCalc increasing by 22.7 versus 10.7 years (*p* = 0.3), respectively (Supplementary Figure 2l). While these differences were not significant, the RNAAgeCalc further substantially overestimated chronological age in iNs (+31.9 ± 3.1 years), while it was more accurate in fibroblasts (−10.7 ± 5.4 years), suggesting that transcriptomic clocks are sensitive to cell‐type specific transcriptomic signatures. We therefore applied the brain‐specific RNAAgeCalc (Ren and Kuan 2020), which yielded age estimates closer to chronological donor age in iNs (−9.5 ± 11.5 years) (Figure 2m). Importantly, this clock detected a pronounced increase in transcriptomic age in iNs derived from 6‐month pre‐aged fibroblasts, corresponding to an average estimated age gain of 21.5 years (Figure 2m–o, Supplementary Figure 2k). This accelerated aging signature was similar to the age gain observed in fibroblasts (Supplementary Figure 2m). Together, these data demonstrate that aging signatures acquired by fibroblasts during extended in vitro culture are preserved during the iN conversion process. This approach provides a viable strategy to generate pseudo‐longitudinal iN samples to model progressive neuronal aging in vitro.

### 2.6 Single‐Cell RNA‐Seq Reveals Increasing Cellular Heterogeneity With Age

To further investigate how these aging signatures manifest across individual cells, we next explored the heterogeneity of the aging process using single‐cell RNA‐seq (scRNA‐seq). While the biological aging process of higher organisms is an inherently heterogeneous process, the heterogeneity of the underlying cellular age, and how it changes during in vitro aging, remains unknown (Işıldak et al. 2020; Bahar et al. 2006; Martinez‐Jimenez et al. 2017; Angelidis et al. 2019; Vidal et al. 2019; Enge et al. 2017; Song et al. 2025; Zou et al. 2021; Nikopoulou et al. 2019). We performed scRNA‐seq of fibroblasts from two donors, aged 67 years (donor #3) and 68 years (donor #4), at Month 0 and following 5 months of in vitro aging (Figure 3a,b). The samples contained 5419–8145 cells per sample that consistently were covered with 23,800–32,500 reads per cell (Supplementary Figure 3a,b). The virtual absence of cells in G2/M phase further confirmed the non‐proliferative state of fibroblasts during long‐term confluent culture in all four samples (Figure 3a, Supplementary Figure 3c). The two donors and time points clearly clustered separately in the UMAP space (Figure 3a,b). We first assessed the level of consistency between transcriptomic ages (RNAAgeCalc—across‐tissue) calculated from scRNA‐seq pseudobulk expression data of the four clusters and compared to the bulk RNA‐Seq results from the same donors and time points. For both donors, predicted transcriptomic age was generally similar, differing by up to 5 years between methods (Figure 3c,d). Only Month 5 of donor #3 showed a notable overestimation of 19 years in single‐cell compared to the bulk RNA‐seq analysis. From the 5419–8145 cells, we randomly pooled 50 single cells per sample into a total of 109–163 micropools for transcriptomic age estimation. Although micropools inherently exhibit variability in gene expression, we observed a consistent shift toward increased transcriptomic age in samples cultured for 5 months (Supplementary Figure 3d). Importantly, in vitro aging robustly captured the biological aging trend and direction across methods and experiments, demonstrating the robustness and reproducibility despite methodological variability.

**FIGURE 3:**
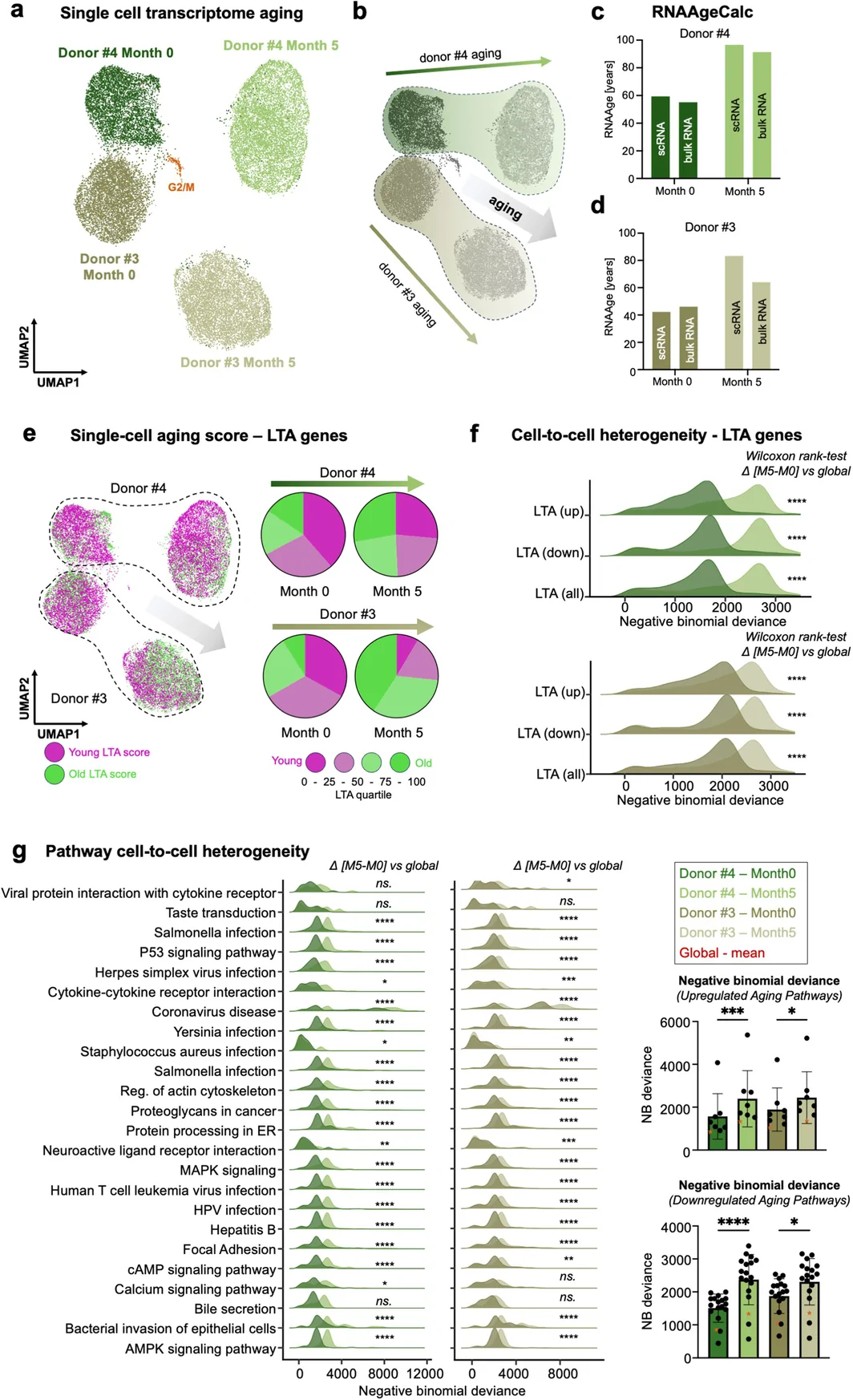
Single cell RNA sequencing shows heterogeneous aging in vitro. (a, b) UMAP of scRNA‐seq of Donors #3 and #4 at Months 0 and 5. (c, d) RNAAgeCalc—across‐tissue of the donors at Months 0 and 5 of bulk RNA‐seq and pseudobulk of scRNA‐seq. (e) UMAP of LTA score based on overlapping aging genes in vitro and in vivo (Supplementary Table 2), dividing cells into young cells (lower 50% LTA score, purple) and old cells (upper 50% LTA score, green). (f) Gene‐level negative binomial deviance per donor and time‐point, reflecting cell‐to‐cell expression variability. Ridge plots show distribution of gene‐level deviance across gene sets. Levene's test for homogeneity of variance was used to compare baseline (M0) and Month 5 of in vitro aging (#4 df = 1, *F* = 8.4, *p* = 0.004; #3 df = 1, *F* = 191, *p* < 2.2e^−16^), and Wilcoxon rank‐sum test comparing Δslope (M5 − M0) between LTA pathway genes (*n* = 718) and background genes (*n* = 24,385) showed a significant enrichment in LTA genes (all: *W* = 20,202,112, adjusted *p* = 1.85e^−139^, up: *W* = 9,937,924, adjusted *p* = 1.39e^−71^, down: *W* = 12,251,977, adjusted *p* = 1.27e^−85^). (g) Ridgeplot representing negative binomial deviance of genes contributing to upregulated (*n* = 7) and the downregulated (*n* = 17) pathways for Donor #3 and #4. Average deviance per pathway, as well as average global deviance (red) was plotted as bar graphs. Normality assumptions were not met and differences were assessed using Friedman test for paired measurements (upregulated pathways: *X* ^2^ = 22.2, df = 3, #4 M0 vs. M5 *p* = 0.001, #3 M0 vs. M5 *p* = 0.01; downregulated pathways: *X* ^2^ = 44.5, df = 3, #4 M0 vs. M5 *p* < 0.0001, #3 M0 vs. M5 *p* = 0.03). (f,g) Significance: *p < 0.05, **p < 0.01, ***p < 0.001, ****p < 0.0001.

We next sought to assess heterogeneity of aging pathways in our cultures. Using Seurat, we calculated single cell aging scores based on the LTA genes, composed of all genes contributing to the overlapping in vitro and in vivo aging pathways (Supplementary Tables 1 and 2). At baseline, most cells from both donors contributed to the youngest quartile of the aging score (39% and 33%), and only 15% and 9% of cells contributed to the upper quartile of old cells (Figure 3e). Following 5 months of in vitro aging, the ratios shifted toward smaller proportions of younger cells and higher proportions of older cells, resulting in 28% (donor #4) and 41% (donor #3) of cells contributing to the oldest quartile (Figure 3e), indicative of a global shift toward older cellular ages in both donors during in vitro aging. To assess cell‐to‐cell heterogeneity, we calculated the negative binomial (NB) deviance, which accounts for mean–variance dependence and other technical factors inherent in scRNA‐seq data. Increasing transcriptional and epigenetic noise and cell‐to‐cell variations are a central theme of biological aging and are now recognized as a hallmark of aging (Işıldak et al. 2020; Bahar et al. 2006; Martinez‐Jimenez et al. 2017; Angelidis et al. 2019; Vidal et al. 2019; Enge et al. 2017; Song et al. 2025; Zou et al. 2021; Nikopoulou et al. 2019). We observed that at Month 5, NB deviance of LTA genes was significantly elevated, indicating a progressive loss of transcriptional control and accumulation of transcriptional noise (Figure 3f). Importantly, this increase exceeded what would be expected based on the global transcriptome, suggesting that the LTA aging‐associated genes are particularly prone to increased heterogeneity (Figure 3f). Heterogeneity increased in both down‐ and up‐regulated aging pathways, with cell‐to‐cell variability significantly rising after 5 months of in vitro aging (Figure 3f,g). To account for potential selection bias from using gene sets identified in our system, we cross‐validated our findings in independent datasets (Supplementary Table 2). First, we calculated single‐cell aging scores using only significant aging genes identified in our in vitro aging system, which mirrored the age‐associated patterns observed with the LTA aging score (Supplementary Figure 3e). Next, we applied two independent aging gene sets: those defined by the RNAAgeCalc and the Fleischer et al. dataset (Supplementary Figure 3f–h) (Işıldak et al. 2020; Bahar et al. 2006; Martinez‐Jimenez et al. 2017; Angelidis et al. 2019). In both cases, we observed the same patterns as with the LTA score, indicating that this phenotype is a general feature of aging genes and not driven by circularity in gene selection. These findings suggest that in vitro aging recapitulates key features of in vivo aging, evidenced by increased aging scores across multiple independent gene sets and a consistent, robust increase in cell‐to‐cell heterogeneity.

### 2.7 Metformin, but Not Rapamycin, Significantly Alters Aging Trajectories

We next examined how anti‐aging treatments influence these aging pathways. Metformin and Rapamycin are often viewed as the two most promising anti‐aging drugs, as they extend lifespan in various animal models (Martin‐Montalvo et al. 2013; Juricic et al. 2022; Park and Shin 2017; Gillespie et al. 2019; Wang et al. 2017; Horvath et al. 2019). In humans, Metformin has been shown to slow epigenetic clocks in diabetic patients, but its effects in healthy individuals remain unclear. Rapamycin extended lifespans in animal models, but has not yet demonstrated clear anti‐aging effects in humans. This underscores the urgent need for predictive, versatile, and cost‐efficient human aging models to evaluate candidate therapeutics (Li et al. 2022; Lee et al. 2024; Strong et al. 2016; Mohammed et al. 2021). To assess predictive power of our accelerated in vitro aging model, we long‐term treated cultures of four donors (Supplementary Figure 1b) with 25 nM Rapamycin or 1 mM Metformin over 6 months and performed RNA‐seq and DNAm analysis each month. No apparent cell death or morphological changes were observed under these conditions (Figure 4a). Interestingly, Metformin treatment had a slowing effect on the DNAm and transcriptomic aging velocity, significantly reducing the average DNAm aging velocity of these donors from 8.23× to −2.8×, and the velocity based on RNAAgeCalc from 48.3× to 11.0× (Figure 4b, Supplementary Figure 4a,c). Given the limited power of paired *t*‐tests in small sample sizes, we further validated these findings using a linear mixed‐effects model. This model revealed a significant interaction effect (*p* < 0.05) of Metformin, confirming that Metformin slows down the rate of DNAm aging. Although Metformin did not show a clear effect on aging velocity assessed by the transcriptome‐wide tAge (Supplementary Figure 4d), it did show a significant reduction of tAge years aged in 5 months, similar to observations in DNAm age (Figure 4c, Supplementary Figure 4d). Rapamycin also showed a slowing trend on both DNAm and transcriptomic clocks, but not at statistical significance (Supplementary Figure 4a–d). To assess the treatment‐specific impacts on the dynamic transcriptional aging process, we performed linear regression analysis including an interaction term between treatment (Metformin or Rapamycin) and time in vitro. This analysis identified 151 aging genes significantly impacted by Metformin treatment after correcting for multiple testing, while Rapamycin affected only 9 genes (Figure 4d, Supplementary Figure 4e, Supplementary Tables 3 and 4). We next focused on the 24 transcriptomic aging pathways that overlap between in vivo and in vitro aging, and found that Metformin significantly attenuated the decline of all downregulated aging pathways, and also impacted upregulated pathways, albeit with a smaller effect size (Figure 4e,f). Effect size was determined based on the difference of baseline and treatment slopes. Metformin almost completely (> 50%) reversed all downregulated pathways and reduced the slope of one‐third of upregulated pathways by > 50% (Figure 4g, Supplementary Figure 4i). In contrast, Rapamycin had minimal effects on either downregulated or upregulated aging pathways. Most pathways showed no significant response to Rapamycin or only a modest rescue of up to 50% for upregulated pathways (Supplementary Figure 4f–i). Overall, our in vitro results align with compelling evidence from pre‐clinical studies, including cynomolgus monkeys showing Metformin's beneficial effects on aging‐related readouts, including epigenetic clocks, although corresponding human trial data remain limited (Yang et al. 2024). This indicates that prolonged treatment of in vitro aging fibroblasts can serve as an evaluation platform for testing potential new anti‐aging compounds relevant to human aging on a personalized level. The approach further appears powerful to identify aging pathways that are refractory to specific treatments to inform about potential combinatorial approaches targeting these missed pathways.

**FIGURE 4:**
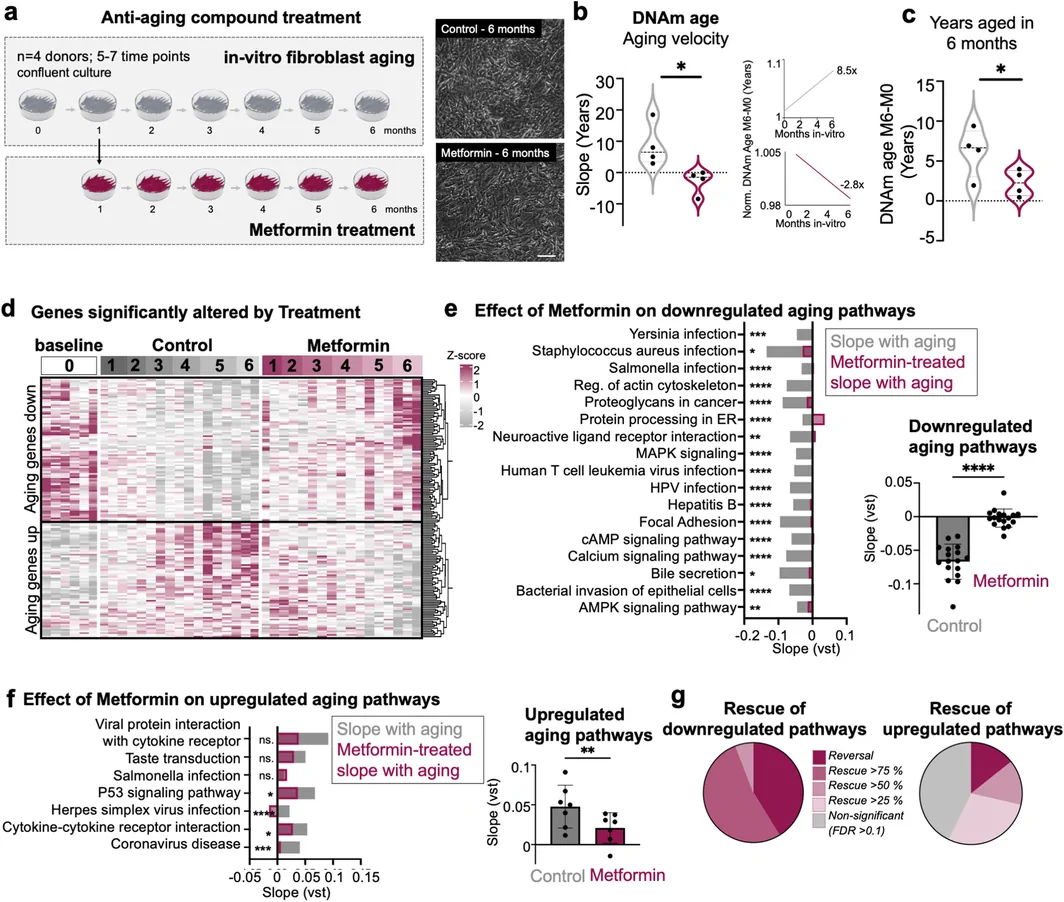
Anti‐aging drug Metformin to slow aging in vitro. (a) Fibroblasts at confluency were treated with Metformin (1 mM) for 6 months (*n* = 4 independent donors). No overt morphological differences were observed. Cells were harvested each month (30 days) for RNA and DNA extraction to assess DNAm age (Blood&Skin clock) and transcriptomic age (RNAAgeCalc—across‐tissue). (b) Slopes of DNAm Blood&Skin clock in Control (gray) and Metformin‐treated (red) cultures (*n* = 4/group). Statistical comparison was performed using a mixed linear effect model (treatment × time in vitro: Estimate = −0.18 ± 0.09, *t*(49) = −2.1). Average simple linear regression slopes across all donors were plotted. (c) Years aged in vitro was calculated by subtracting the age of Month 0 from Month 6 in Control (gray) and Metformin‐treated cultures (red). Paired *t*‐test was performed (*t*(3) = 4.4, 95% CI [−20, −3], *p* = 0.02). (d) Mixed‐effects linear model was performed. The heatmap represents all significant genes (FDR < 0.1) for the interaction term of treatment with time in vitro. Genes are shown at baseline, after in vitro aging (left, gray), and after Metformin treatment (right), scaled per row. (e, f) Effect of Metformin on aging pathways. Gray bars represent baseline slope of linear regression with time in vitro (as years, vst‐normalized), red bars represent the treatment slope. A red slope of 0 indicates that Metformin fully (100%) rescues the aging pathway, and is no longer up‐ or downregulated. For each pathway, gene‐wise baseline slopes and treatment interaction slopes were extracted and compared using paired *t*‐test (adjusted for multiple testing, FDR). Average slopes per pathway were plotted as a bar graph (Paired *t*‐test, upregulated pathways *t* = 3.9, df = 6, *p* = 0.0076; downregulated pathways *t* = 13.8, df = 16, *p* < 0.0001). (g) Percent rescue defined as the ratio of the mean interaction slope to the mean baseline slope (−Mean baseline slope/Mean Interaction slope) * 100. Significance: *p < 0.05, **p < 0.01, ***p < 0.001, ****p < 0.0001.

## 3 Discussion

Collectively, this longitudinal transcriptomic and DNAm characterization of in vitro aging in human fibroblasts from adult donors provides novel insights into the biological aging process during extended periods of in vitro cell cultures. By comparing identical cell types in vivo and in vitro, our findings reveal that the aging process in vitro reflects many aspects of transcriptomic and DNAm aging. We observe a pronounced acceleration of aging clocks within the in vitro system, consequently allowing us to replicate multi‐year time frames within just a few months in culture. This presents a unique opportunity to simulate the crucial time period preceding the onset of age‐related diseases (Farias et al. 2009). Notably, the observed increase in DNAm and transcriptomic age during long‐term in vitro cultures occurs independently of cellular senescence. Our data reinforce and extend previous observations that the progressive increase of biological age during prolonged in vitro culture represents a molecular process distinct from replicative senescence (Kabacik et al. 2022). The observation that transcriptomic aging estimates increased ~5‐fold faster than DNAm age estimates suggests an apparent uncoupling of transcriptional and DNAm aging trajectories during prolonged in vitro culture. This disconnect was reproducibly detected across independent transcriptomic clocks, indicating that it is not driven by the assumptions or gene set of a single estimator. This implies that transcriptional aging programs may be more plastic and hypersensitive to the loss of tissue‐level homeostatic constraints, whereas DNAm aging may accumulate more gradually over time. Consistent with this distinction, transcriptomic clocks trained on different gene expression signatures may capture partially distinct aspects of the aging process, resulting in greater variation in age estimates across clocks and cell types compared with DNAm clocks. Importantly, however, the consistent increase in transcriptomic age across multiple independent clocks in our study supports the robustness of the observed accelerated transcriptomic aging, despite differences in the magnitude of age acceleration between estimators. Because transcriptomic and DNAm clocks quantify distinct molecular features using different methods, the observed difference in clock velocities should be interpreted as a relative divergence in these aging readouts, rather than as definitive evidence that the underlying transcriptomic and epigenetic processes are mechanistically independent. As a donor‐specific model of accelerated aging, this fibroblast in vitro system enables the assessment of pharmacological interventions targeting aging processes other than senescent cell buildup. Because confluent fibroblast cultures are contact‐inhibited, they can be cultured for months in a non‐dividing G0‐like state that circumvents senescence. Further, single cell transcriptome analysis detected a substantial heterogeneity of cell ages within primary cell populations. A similar phenomenon has been observed in vivo (Işıldak et al. 2020; Bahar et al. 2006; Martinez‐Jimenez et al. 2017; Angelidis et al. 2019), and our data further exemplifies that the increase of biological noise is a multi‐parallel and defining theme of biological aging. Our single cell data extend this notion from intra‐cellular noise (transcriptomic, epigenetic) to inter‐cellular heterogeneity within a population. Nevertheless, dedicated studies systematically examining transcriptional heterogeneity during fibroblast in vivo aging remain limited. A potential limitation of our heterogeneity analysis lies in the inherent heteroscedastic nature of age predictors, particularly trained clocks, where prediction variance increases with age. This could partially contribute to the observed cell‐to‐cell variability in our single‐cell data.

Furthermore, our data have significant implications for enabling dynamic aging research using direct reprogramming models, such as iNs that retain the aging signatures of their original cell types (Pitrez et al. 2024; Mertens et al. 2021; Traxler et al. 2022; Herdy et al. 2022; Huh et al. 2016). Because iNs, unlike iPSCs, retain the aging characteristics of their starting cells, we have demonstrated a novel approach to generate pseudo‐longitudinal aged neuronal samples by applying our in vitro aging model to human fibroblasts and subsequently converting them into iNs. This method provides a valuable tool for studying the progressive effects of aging on neurons, potentially accelerating research into age‐related neurodegenerative disorders and the development of therapeutic interventions. While a 5‐year increase in DNAm aging on average does not recapitulate decades‐long biological aging that precedes neurodegenerative disease, it enables investigation of molecular mechanisms happening in a defined time window prior to clinical disease onset. This may include the progression from mild cognitive impairment (MCI) to Alzheimer's disease (AD), which has a 13% annual conversion rate (Farias et al. 2009), or assessment of pre‐disease molecular changes in induced motor neurons from carriers of familial ALS mutations, allowing study of the trajectory from pre‐symptomatic to disease onset (Westeneng et al. 2025; Buon et al. 2025; Stikvoort García et al. 2025).

Collectively, our findings suggest that in vitro aging of starting fibroblasts can be employed to “pre‐age” these cells prior to neuronal conversion to generate “pseudo‐longitudinal” neuron samples from a single donor, particularly in scenarios where longitudinal biopsies are not feasible.

## 4 Limitations of the Study

This manuscript is primarily aimed at evaluating the extent to which a long‐term in vitro culture model can recapitulate key features of human aging, rather than identifying novel aging pathways. Likely sources of pathway‐level and gene‐level variability may be due to donor demographics, cohort size, and analytical methods. Furthermore, while we employed statistical models (negative binomial deviance) to account for technical noise in our single‐cell heterogeneity analysis, we cannot fully exclude the contribution of stochastic transcriptional bursting or amplification artifacts inherent to scRNA‐seq. While our in vitro aging model offers valuable insights, it is important to recognize that it does not fully replicate the complexities of real longitudinal studies. For instance, our approach does not account for the intricate environmental factors that influence aging in vivo, such as diet, exercise, and exposure to various stressors. Additionally, it cannot capture patient‐specific events during the aging process, including illnesses or lifestyle changes, which may significantly impact individual aging trajectories. Despite these considerations, fibroblasts provide a robust starting point for several direct conversion protocols to various cell types. Currently, our method is primarily focused on generating neurons; however, there is great potential for further development. By expanding direct conversion protocols to include alternative cell types, such as glial cells, we can enhance the capabilities of our model system. This expansion will allow for a more comprehensive exploration of cellular aging across different tissue types and contribute to a deeper understanding of age‐related processes. In this study, we tested one concentration of anti‐aging compounds Rapamycin and Metformin as a proof of concept. Doses were chosen based on literature as the minimal dose that showed effects before to avoid toxicity of long‐term treatments. Dedicated studies are required to assess the optimal dosage.

## 5 Materials and Methods

### 5.1 Experimental Model and Subject Details

The cohort consists of four fibroblast lines derived from all female donors between 53 and 68 years (Supplementary Figure 1b). All experiments conducted were within the approval of the institutional ethics committee. Human skin samples were obtained with the ethical approval of the ethics committee of the Medical University of Innsbruck (EK Nr: 1053/2018), and informed and signed consent was obtained from the donors before collection of tissues.

### 5.2 Isolation and Culture of Primary Human Skin Fibroblasts

Primary cells were isolated from skin punch biopsies, which were immediately digested using 1 mg/mL Collagenase and 1 mg/mL Dispase at 37°C overnight, before enabling outgrowth of fibroblasts on Gelatin‐coated wells. Pure fibroblast cultures were generated from four donors (female, 53, 55, 67 and 68 years old) and maintained in fibroblast media containing DMEM (ThermoFisher) + 15% fetal bovine serum (ThermoFisher) + 1× non‐essential amino acids (Sigma Aldrich) + 1× antibiotic‐antimycotic (ThermoFisher). Cells were expanded by detaching using TrypLE (ThermoFisher) and seeded in a 1:2 ratio. Month 0 of the experiment was defined as the time point at which all donor lines were available in 18 confluent flasks. Passage numbers for each donor at this time point are provided in Supplementary Figure 1b. Cells at the Month 0 timepoint were detached upon reaching confluency using TrypLE, then pelleted and snap‐frozen on dry ice, and stored at −80°C. For the Months 1–6 timepoints, full media changes were performed twice per week. One flask per cell line was harvested every 30 days for up to 6 months. In vivo aging fibroblasts (passage < 10) and in vitro aging fibroblasts were harvested once reaching confluency using TrypLE and pelleted at 4°C. Pellets were stored in liquid nitrogen until processing. In vitro aging fibroblasts were harvested each month (30 days) for 6 months. Metformin (1 mM) and Rapamycin (25 nM) treatments were applied the day after harvesting Month 0 in fibroblast media. Media including compounds were freshly prepared for each media change and continuously applied to the cells throughout the 6‐month culture period.

### 5.3 DNA/RNA Extraction

Frozen cell pellets of Months 0–6 were thawed on ice, and DNA and RNA were extracted simultaneously from the same pellet using the Qiagen AllPrep DNA/RNA Mini Kit. DNA and RNA concentrations were assessed using Qubit fluorometric quantification. Quality of RNA was further assessed using the TAPE Station System (Agilent), and DNA was run on an agarose gel.

### 5.4 Bulk RNA Sequencing

RNA samples were frozen and shipped on dry ice. In vitro aging samples were sequenced paired‐end 150 bp on the NovaSeq X Plus at 20 million paired reads per samples (NovoGene) and the in vivo aging samples were sequenced paired‐end 150 bp on the NextSeq at 20 million paired reads per sample. Read trimming was performed using TrimGalore, and reads were aligned to the hg38 reference genome using STAR. Gene‐level raw counts were generated with featureCounts. Additional raw count data from the RNA‐seq study of aging fibroblasts by Fleischer et al. were obtained from NCBI Gene Expression Omnibus (GSE113957). Where gene identifiers were provided as ENSG IDs, they were converted to official gene symbols using BioMart. Identical preprocessing steps were applied across all datasets. This included DESeq2‐based quality control, removal of LOC and miRNA genes, exclusion of genes with missing values, and filtering for expressed genes using a threshold of > 0.5 CPM. Only genes that passed these filtering criteria and were present in all three datasets were retained. Variance‐stabilizing transformation (vst) was applied, followed by linear regression analyses using limma. For the in vitro aging dataset, all RNA‐seq samples collected between Months 0 and 6 were included in a linear regression analysis with culture time as a continuous variable. Donor ID was included as fixed effect and repeated measures were accounted for using duplicateCorrelation. For the in vivo aging datasets, all available samples spanning the indicated age ranges (22–92 years for our in vivo cohort, 1–96 years for the Fleischer et al. cohort) were analyzed using age as a continuous variable, with sex included as a covariate (model = age + sex). Genes showing a significant association with age (adjusted *p*‐value < 0.05 for in vitro and Fleischer et al., *p*‐value < 0.05 for in vivo dataset) were termed “aging‐genes”.

### 5.5 Pathway Analysis

KEGG pathway enrichment analysis was performed using ClueGO (Cytoscape) (Bindea et al. 2009) on lists of significant age‐associated genes identified from linear regression analyses. Separate analyses were performed for positively and negatively age‐associated genes. For each dataset, genes were assigned to these lists according to the sign (up‐ and down‐regulated) and statistical significance of their regression coefficient for culture time or chronological age. Aging gene lists from the in vitro dataset (all samples collected from Month 0–6), our in vivo fibroblast cohort (all donors aged 22–92 years), and the independent in vivo cohort (all donors aged 1–96 years) were analyzed simultaneously in ClueGO to enable direct comparison and minimize dataset‐dependent biases in pathway assignment. Pathway inclusion cutoffs were defined based on the “adjusted term *p*‐value” smaller than 0.1 and the percentage of genes contributed by each dataset. A threshold of 15% was required for a pathway to be considered overlapping across datasets, with 33% representing an equal contribution from all three datasets and 100% indicating exclusive contribution from a single dataset. Owing to the relatively small number of aging genes identified in the in vivo aging dataset, this threshold was relaxed to a minimum of 1% contribution for pathways to be included as overlapping. To confirm that the pathways were indeed enriched in all aging datasets, we further performed gene‐set enrichment analysis (fgsea, R). Genes derived from ClueGO analysis were used as input per pathway. Aging gene sets were ranked based on *t*‐value (combines slope and *p*‐value). Genes derived from pathways with significant enrichment in all three datasets were termed LTA genes. The complete list of aging genes can be found in Supplementary Tables 1 and 2. Fisher's exact test was performed to test for significant overlap between datasets, using all identified pathways from ClueGO as background.

To test whether aging trajectories differed between datasets, we fit a linear model including an interaction term between age and dataset (PathwayExpr∼Age×Dataset). In this model, the in vitro dataset was used as the reference level, and interaction terms quantify the difference in slope between each in vivo cohort and the in vitro reference. Statistical significance of slope differences was assessed using the interaction term *p*‐values, which were corrected for multiple testing across pathways using the Benjamini–Hochberg procedure. To visualize pathway‐level expression, gene expression values were averaged across all genes within each pathway for each donor, such that each dot represents the mean pathway expression per donor.

For RNA age analyses, some samples from timepoint 6 were excluded due to lower RNA quality. Additionally, we observed a general drop in RNA age at later timepoints across several samples. As this cannot be attributed to sample quality or outlier effects, it may represent a genuine shift in global transcriptional dynamics, potentially linked to yet unknown biological effects. Further mechanistic studies are needed to elucidate the drivers of this non‐monotonic trajectory.

### 5.6 DNA Methylation EPIC Array

DNA methylation assays were performed using the Infinium Methylation EPIC v2.0 Kit, loading 8 samples per BeadChip as per the standard manufacturer's protocol (Illumina). Raw intensity IDAT files were processed and analyzed using the R packages ChAMP and RnBeads, arrays were normalized using the BMIQ procedure. Beta‐values were annotated to the EPICv2.0 annotation file and linear regression and correlation across time was calculated. Promoter regions were defined as the average methylation levels of TSS200, TSS1500, 5′UTR, and 1st exon based on previous reports (Ren and Kuan 2019).

### 5.7 Biological Age Estimators

DNAm Clock analysis was performed using the UCLA online tool “DNA methylation age Calculator” to predict chronological ages of cell culture samples (Horvath 2013).

The multi‐tissue transcriptional age calculator RNAAgeCalc (Ren and Kuan 2020) was used to calculate transcriptomic age. RNA‐seq data of in vitro aging samples were further analyzed using featureCounts to generate Fragments Per Kilobase of transcript per Million mapped reads (fpkm) normalized counts. To assess transcriptomic age of in vitro aged fibroblasts and iNs, we applied the across‐tissue clock trained on 26 different tissues to fpkm normalized counts according to the Bioconductor script. For in vivo datasets, we applied the age calculator to rawcounts using the built‐in normalization. For iNs, we further applied the brain‐specific clock.

RNAge transcriptomic age prediction was calculated in R (v4.5.1) using the source code provided by Zhang et al. at https://github.com/zhangch/RNAgePaper (Zhang et al. 2025). Whole transcriptome expression data were normalized to transcripts per million (TPM) and divided into two groups. For in vitro aging, all four donors at Month 0 were considered the reference group, which was compared to all four donors at Month 6. In in vivo aging, samples from both our in vivo aging cohort and Fleischer et al. were divided into young (< 27 years, *n* = 50) and old (> 67 years, *n* = 50) for the relative age comparison.

tAge transcriptomic age (Tyshkovskiy et al. 2026) was calculated using R (v4.5.1) with the tAge package (v1.0.1) and Python (v3.10.10). tAge was predicted from raw transcriptomic count data using the pretrained model BR_Chronoage_Multispecies_Multitissue_yugenediff.pkl. Raw counts were normalized using the YuGene transformation and age predictions were generated using Baysian ridge regression. Batch‐effect correction using SVA (v3.56.0) and CombatSeq was applied to raw counts of the in vitro fibroblast dataset to adjust for potential batch effects.

### 5.8 Aging Velocities

Aging velocities were defined as the slope of a linear regression with age in years, representing the rate of molecular change per year of in vitro or in vivo aging. Aging velocities were calculated either in GraphPad Prism (simple linear regression, best‐fit slope values) or in R using the limma package. Interaction terms (age × sex) were added where indicated. Age was consistently expressed in years, with 6 months of in vitro aging corresponding to 0.5 years. For transcriptomic data, *p*‐values from regression models were corrected for multiple testing using the false discovery rate (FDR, Benjamini–Hochberg method).

### 5.9 Direct Conversion of Pre‐Aged Fibroblasts Into Induced Neurons

Fibroblasts at starting passage were transduced with an all‐in‐one lentiviral vector for dox‐inducible expression of the neuronal transcription factors (Ngn2 and Ascl1) and selected and expanded under puromycin. At Month 0 (3–4 weeks following lentiviral transduction) and after 6 months, the transgenic fibroblasts were converted to iNs using our previously described protocol (Mertens et al. 2015, 2021; Zhou‐Yang et al. 2021). Briefly, the cells were treated with dox along with small molecule enhancers to promote neuronal identity for 3 weeks. At Day 21, iNs were fixed using 4% paraformaldehyde for immunocytochemistry. For DNA and RNA extraction, iNs were purified using FACS sorting. Cells were dissociated using TrypLE and labeled with PSA‐NCAM antibodies. Fluorescently labeled cells were sorted using FACS to isolate PSA‐NCAM‐positive iNs, ensuring a highly enriched neuronal population (> 90% purity). Sorted cells were immediately pelleted and processed for DNA and RNA collection as described for fibroblasts.

### 5.10 Single Cell RNA Sequencing

Before reaching Month 5 of the experiment, we thawed a previously frozen batch of fibroblasts of Donors #3 and #4, representing Month 0 once reaching confluency. Fibroblasts from Months 0 and 5 were simultaneously harvested for single cell RNA sequencing. Cells were counted in PBS + 0.04% BSA and loaded on the 10× Chromium Controller targeting 8000 cells per sample. Library was prepared using the 10× 3′scRNA v3.1 library kit and 10× Dual Index Kit, and was sequenced on the NextSeq 2000 (200 cycles kit). Raw files were processed using Cell Ranger and analyzed using Seurat. Pseudobulk expression profiles were generated for each cluster and used as input for biological age estimation with RNAAgeCalc. Additionally, random micropools of 50 cells were generated using Seurat. Aging module scores were calculated at the single‐cell level using Seurat. The RNA assay was log‐normalized using Seurat's NormalizeData function, and aging module scores were calculated using Seurat's AddModuleScore function. Separate scores were computed for each aging gene set (LTA, Fleischer, in vitro aging, and RNAAgeCalc; gene list derivation described in the RNA‐seq analysis section). For each cell, AddModuleScore computes the average normalized expression of the genes in the target list and subtracts the average expression of randomly selected control genes matched for similar overall expression levels (100 control genes per target gene; ctrl = 100). This produces a relative per‐cell module score that does not require any additional scaling or normalization to a reference sample or time point. The age scores were calculated by supplying the gene lists as input to AddModuleScore; the resulting per‐cell module score was stored in the Seurat object metadata and used for all downstream analyses.

### 5.11 Negative Binomial Deviance Analysis

Cell‐to‐cell transcriptional heterogeneity was quantified using NB deviance computed directly from raw single‐cell RNA‐seq UMI counts. Raw counts were extracted from the Seurat object (RNA assay, counts slot) and retained in sparse matrix format. No normalization, scaling, or log‐transformation was applied prior to deviance estimation. Size factors were calculated as total UMI counts per cell normalized to the mean library size. For each gene and sample, mean expression and variance across cells were estimated, gene‐specific dispersion parameters were inferred assuming a negative binomial distribution, and NB deviance was computed as a gene‐level measure of excess cell‐to‐cell variability. NB deviance was calculated independently for each sample. Genes contributing to aging scores and genes belonging to predefined LTA pathways were analyzed. Pathway‐level deviance was summarized per sample by computing the mean NB deviance across all genes within each pathway. To assess temporal changes in heterogeneity, gene‐level NB deviance values at Month 0 and 5 were compared within each donor using paired Wilcoxon signed‐rank tests. To test whether pathway‐associated changes exceeded global trends, gene‐level ΔNB deviance (Month 5 − Month 0) for pathway genes was compared to background genes using Wilcoxon rank‐sum tests. *p*‐values were adjusted for multiple testing using the Benjamini–Hochberg procedure.

### 5.12 Immunofluorescence

Fibroblasts were cultured for up to 6 months on 8‐well μ‐Slides (Ibidi, Germany) and fixed once confluent (M0), and after three and 6 months of non‐proliferative culture with two media changes per week in fibroblast media + antibiotics. Fibroblasts were fixed using 4% paraformaldehyde at each time‐point and stored at 4°C. Independent fibroblast lines were passaged until reaching senescence and fixed on 8‐well μ‐Slides. For iN conversion, fibroblasts with inducible expression of Ngn2 and Ascl1 were cultured for 6 months on 8‐well μ‐Slides or split at 6 months onto 8‐well μ‐Slides before starting direct conversion. iNs were fixed using 4% paraformaldehyde after 21 days of conversion at Months 0 and 6. Fibroblasts used as positive control for senescence were seeded on 8‐well Ibidi slides and treated with Etoposide (20 μM) for 48 h before fixation with 4% PFA. Cells were blocked in PBS containing 0.05% Triton‐X100 and 5% FBS and stained in PBS containing 3% FBS using the primary antibodies p21 Waf1/Cip1 (12D1) (Cell Signaling Technology, #2947, 1:500), CDKN2A/p16INK4a (Ab108349, rabbit, 1:500), tubulin‐beta 3 (TUBB3, BioLegend, Clone AA10, 1:1000), NeuN (Cell Signaling Technology, D4G40, 1:400), MAP2 (Abcam, ab5392, 1:400) at 4°C overnight and incubated with secondary antibodies for 2 h at room temperature, followed by 10 min of DNA staining with DAPI (ThermoFisher, D21490, 300 nM). Images were taken at the ECHO Revolve Microscope or z‐stacks at the Dragonfly 200 spinning disk confocal microscope and reconstructed using Bitplane Imaris 8.2 software. Senescent cells were counted and normalized to the total number of cells marked by DAPI.

### 5.13 Calculation of Transcriptomic Effects of Anti‐Aging Compounds

Bulk RNA‐seq raw counts from the control cohort (including one replicate at timepoint 0 of donor #4), as well as transcriptomic data from Metformin‐ or Rapamycin‐treated cells (*n* = 3 per timepoint), were variance‐stabilizing transformed (vst) for normalization. Outliers were identified via principal component analysis (PCA) and excluded from further analysis. To assess treatment‐specific effects over time, linear regression models including an interaction term between time‐in‐culture and treatment were applied (time × treatment). Genes with a significant interaction effect were based on a false discovery rate (FDR) threshold of < 0.1. For downstream analysis, the slopes corresponding to pathway genes were extracted and averaged to quantify pathway‐specific treatment effects. The percent rescued was calculated using (− Mean baseline slope/Mean Interaction slope) * 100. This approach defines a 100% rescue when the treatment slope equals zero (complete rescue to a flat trajectory), and 0% rescue when the treatment slope is identical to the baseline slope (no rescue). For each pathway, a gene‐wise paired *t*‐test was performed to test whether treatment slopes differ significantly from the baseline slope. *p*‐values were adjusted for multiple testing using FDR.

We analyzed the longitudinal effects of anti‐aging compounds on aging trajectories using a linear mixed‐effects model implemented with the nlme and lme4 R packages. The model included fixed effects for time, treatment, and their interaction to test whether the compounds altered the rate of change over time. Subject‐specific random intercepts were included to account for baseline differences between donors. Missing data points were excluded via listwise deletion (na.omit). The key test of interest was the time‐by‐treatment interaction, which assesses whether the slope of aging differs between treatment and control conditions.

### 5.14 Senescence RNA‐Seq Analysis

SASP factors were selected based on published reviews (González‐Gualda et al. 2021) and expression was quantified from RNA‐seq data. For each donor, expression values (fpkm) were normalized to the baseline value (Month 0), and heatmaps were scaled per row. As a positive control, RNA‐seq data from proliferative and senescent BJ fibroblasts (GSE63577) (Marthandan et al. 2016) were included. The published dataset was analyzed independently to identify SASP factors induced upon replicative senescence within that study and was used as a qualitative reference only; no direct quantitative comparison of gene expression was performed between the published dataset and our in vitro aging dataset. Because the BJ fibroblast dataset was not integrated with our dataset for downstream analyses, no batch correction or cross‐dataset normalization was performed. The Gene‐set enrichment analysis (GSEA) desktop app (version 4.4.0) was used to assess enrichment of senescent terms in in vitro aging.

### 5.15 Statistical Analysis

Normality of data was assessed using the Shapiro–Wilk or Kolmogorov–Smirnov tests. Two‐tailed statistical tests were used unless otherwise stated. Bar plots display mean ± standard deviation; violin plots show the median and interquartile range. If sample size was > 1 and less than 100, individual data points are shown as dots. Statistical significance is indicated as follows: *p* < 0.05 (*), *p* < 0.01 (**), *p* < 0.001 (***), and *p* < 0.0001 (****). ANOVA and *t*‐tests were performed using GraphPad Prism. Linear regression and correlation analyses were performed using GraphPad Prism and RStudio.

## Author Contributions

L.T. and L.K. contributed equally to this study. L.T, L.K., H.I.D.M., F.H.G., and J.M. designed the study, L.T., L.K., T.O., K.C., A.S., L.B. conducted tissue culture work, M.D. and B.C.B. acquired patient samples, J.R.H. and L.Z.‐Y. performed senescence analysis, L.T. and L.K. extracted nucleic acids and sequenced samples, L.T., L.K. and O.B. performed bioinformatic analyses and visualized data, L.T. and J.M. wrote the paper. All have thoroughly discussed this project and helped improve the paper.

## Funding

This work was supported by the National Institutes of Health (R01AG056306, R01AG085634, P30AG062429, U19AG023122, R37AG072502, R01NS135607), BrightFocus Foundation (A2024036S, A2019562S, A2022024F), Alzheimer's Association (AARG‐22‐972303), American Federation for Aging Research (AFAR‐30376715), Live Like Lou Foundation, and California Institute for Regenerative Medicine (EDUC4‐12804).

## Conflicts of Interest

The authors declare no conflicts of interest.

## Supporting information


**Supplementary Table 1:** CLUEGO pathways in vitro aging.

**Supplementary Table 2:** Genes contributing to single cell scores.

**Supplementary Table 3:** Effects of Metformin on age‐associated gene expression.

**Supplementary Table 4:** Effects of Rapamycin on age‐associated gene expression.

**Supplementary Figure 1:** Aging clocks in vitro and in vivo. (a) Overview of fibroblasts used in in vivo aging. (b) Overview of starting fibroblasts for in vitro aging. (c) Immunofluorescence staining (p16) of fibroblasts at Month 0 and 6. Positive control represent senescent fibroblasts (Etoposide, 20 μM, 48 h). (d) Quantification of nuclear Area in fibroblasts undergoing replicative senescence (passage 30+), and at Month 0, 3, and 6 of non‐replicative in vitro aging. One‐way ANOVA (*F*(3, 10) = 11.3, *p* = 0.0015). (e) Gene expression of major SASP factors (scaled per row, vst normalized) in an independent dataset of proliferative and senescent fibroblasts (GSE63577, Marthandan et al. 2016) and non‐replicative in vitro aging (Month 0–6). Average expression (vst‐normalized) of all SASP factors per donor and timepoint are represented as dots above heatmap. (f) DNAm age was calculated using the Online New Methylation age Calculator for the in vivo cohort of donors between 0 and 92 years of age (*n* = 22). Pearson correlation of chronological age and estimated age calculated using Blood&Skin DNAm age, Hannum clock and PhenoAge calculator. (g) Clock accuracy defined as the difference between estimated age and chronological age for DNAm clocks. Repeated measures ANOVA (*F*(1.6, 24.0) = 50.45, *p* < 0.0001). (h–k) Calculation of transcriptomic age in our small in vivo dataset (*n* = 20, 1a) and the Fleischer et al. aging dataset. Pearson correlation was calculated on each dataset individually and after merging both datasets. (h) RNAAgeCalc—across‐tissue (i) RNAAgeCalc—fibroblast‐specific (j) tAge and (k) the transcriptomic age of Peters et al. (2015). (l) Relative transcriptomic aging clock on merged in vivo and Fleischer et al. datasets comparing donors ≤ 26 years or ≥ 68 years (*n* = 50 per group), and on the in vitro aging dataset comparing baseline (M0) and 6 months of in vitro aging (M6). (m) Linear mixed effect model (DNAm~Age~(1|Donor)) of Blood&Skin DNAm Age. Simple Linear Regression was performed to assess slope. (n) DNAm age at baseline (M0) and after 6 months of in vitro aging (M6). Each dot represents an independent donor. Paired *t*‐test (*t*(3) = 3.3, 95% CI [0.22, 10.7]). (o) RNAAgeCalc—across‐tissue of the in vitro aging transcriptomic dataset (*n* = 4 independent donors). Blue line represents slope of same transcriptomic clock based on in vivo aging (small cohort). Linear mixed effects model on RNAAgeCalc—across‐tissue. Residual age was calculated by subtracting the chronological age from the RNAAge. (p) RNAAgeCalc—across‐tissue at baseline and at Month 5 of in vitro aging. Paired *t*‐test (*t*(3) = 4.375, 95% CI [5.9, 37.2]). tAge at baseline and at Month 6 of in vitro aging. Paired *t*‐test (*t*(3) = 2.6, 95% CI [−6.5, −59.8]). Each dot represents an independent donor.
**Supplementary Figure 2:** Transcriptomic changes during in vitro aging. (a) Linear regression of in vitro aging (adjusted *p* < 0.05), in vivo aging (*p* < 0.05) and Fleischer et al. (adjusted *p* < 0.05) across age/time in vitro. Overlap of significant genes. (b) Up‐ and downregulated in vitro aging genes were ranked by slope (logFC, limma) and tested for enrichment in in vivo datasets, and vice versa. Similarly, in vivo aging gene rankings were tested across independent in vivo datasets, demonstrating strong reciprocal enrichment. (c, d) Significant genes from all three datasets were included in a shared pathway analysis using ClueGO with a common ontology‐based network and identical statistical parameters. Pathways with significant term *p*‐values are shown; dot size represents the percentage contribution of the aging gene set to each pathway. All identified pathways were further tested for enrichment using fgsea (R), and the dot color corresponds to the −log_10_(*p*‐value) from the fgsea analysis. (e, f) Vst‐normalized expression was averaged per pathway (overlapping pathways from Figure 2b), and linear regression was used to estimate pathway‐level slopes (vst‐normalized counts). Statistical comparison of slopes was performed in R. To assess pathway‐level differences between in vitro and in vivo aging at gene‐level, we fit a linear model including an interaction term between age and dataset (Age × Dataset) with in vitro aging as reference. Interaction *p*‐value was corrected for multiple testing using the Benjamini–Hochberg method. (g) Linear regression slope across in vitro aging (green), in vivo aging (blue), or Fleischer in vivo aging (brown) of three representative accelerated KEGG pathways in vitro, each dot representing the slope of a gene contributing to this pathway. (h) Box plot of slopes (vst‐normalized counts, years) across all genes in the dataset. (i) GSE113957 divided into young (< 40 years, *n* = 67) and old (> 40, *n* = 76). Pearson correlation of slopes (vst‐normalized, years) after removal of 1.5 IQR outliers (linear regression, adjusted for sex). (j) Fibroblasts with inducible expression of Ascl1 and Ngn2 were maintained in non‐proliferative culture for up to 6 months before conversion induction (doxycycline). Transcriptomic age was assessed at Day 21 after conversion induction (*n* = 3 independent donors) in iNs at baseline (M0) and after 6 months of in vitro aging (M6). RNAAgeCalc—across‐tissue. Years aged was calculated by subtracting RNAAge at baseline from Month 6. Unpaired *t*‐test (*t*(9) = 3.2, 95% CI [2, 12]). (k) DNAm (*n* = 3 at M0, *n* = 2 at M6) and transcriptomic age clocks (*n* = 3 per group) were assessed in iNs generated from replated fibroblasts. Fibroblasts were replated at baseline (M0) or after 6 months of pre‐aging (M6) before direct conversion to iNs. Paired *t*‐test. Each dot represents an individual donor. Lines connect donors at different timepoints. (l‐m) Years aged in iNs and fibroblasts from the same donors (*n* = 3) as calculated by subtraction of estimated age at baseline from age at Month 6 (Month 5 in RNAAgeCalc—across‐tissue). Paired *t*‐test. Average estimated age per group is indicated in the graph.
**Supplementary Figure 3:** Single cell RNA sequencing of in vitro aging. (a, b) Cell number and reads per cell for each donor (*n* = 2) at Months 0 and 5. (c) Proportion of cells contributing to each cluster, showing a clear annotation of each donor at each time‐point. Each donor shows a minimal and comparable number of cells in G2M phase. (d) RNA age calculator age prediction for scRNA‐seq micropools (50 cells per micropool) analyzed using Seurat. Ordinary one‐way ANOVA and Sidak's multiple comparison test (*F*(3, 537) = 225, *p* < 0.0001, #4 M0 vs. M5 *p* < 0.0001, #3 M0 vs. M5 *p* < 0.0001). (e–g) Aging score calculated using Seurats AddModuleScore, based on genes extracted from in vitro aging linear regression (adjusted *p* < 0.05), the RNA age calculator—no tissue, and Fleischer et al. linear regression (adjusted *p* < 0.05) (Supplementary Table 2). Cells in the lower 50% quartiles were considered young (purple), and cells contributing to the upper 50% were considered old (green). Pie charts show a quantification of the number of cells contributing to each quartile. (h) Negative binomial deviance representing cell‐to‐cell heterogeneity of aging scores. Wilcoxon rank‐sum test revealed a significant difference in NB deviance between M0 and M5 (*p* < 0.0001). Wilcoxon rank‐sum test comparing Δslope (M5 − M0) between aging pathway genes and background genes showed a significant enrichment in aging pathway genes (*p* < 0.0001).
**Supplementary Figure 4:** Anti‐aging drug Rapamyn showing limited effects on human cell aging (a) DNAm Blood&Skin clock in Control (gray), Metformin‐treated (red) and Rapamycin‐treated (yellow) cultures. Simple linear regression, comparing slopes. Statistical comparison was performed using a mixed linear effect model (treatment × time in vitro: estimate = −0.18 ± 0.09, *t*(49) = −2.1). (b) Aging velocity based on DNAm age per donor (in years; Paired *t*‐test: *t*(3) = 1, 95% CI [−30, 16], *p* = 0.39). Years aged in vitro calculated by subtracting the age of Month 0 from Month 6 in Control (gray) and Rapamycin‐treated cultures (yellow). (Paired *t*‐test: *t*(3) = 1.0, 95% CI [−10, 5], *p* = 0.38). (c) RNAAgeCalc—across‐tissue on Control (gray), Metformin‐(red) and Rapamycin‐(yellow) treated cultures. Simple linear regression was performed to determine difference of slopes. Violin plots of slopes (in years) and years aged in vitro, repeated measures one‐way ANOVA (*F*(2) = 6.7, *p* = 0.06) and Sidak's multiple comparisons test (Control vs. Metformin *p* = 0.055, Control vs. Rapamycin *p* = 0.37). (d) Effect of anti‐aging treatment on tAge. Repeated measures ANOVA with Dunnet's multiple comparisons test. Velocity: *F*(1.1, 3.3) = 0.04, *p* = 0.86. Years aged: *F*(1.4, 4.11) = 6.0, *p* = 0.06). (e) Mixed‐effects linear model was performed. The heatmap represents top 100 genes (sorted by smallest FDR, including 9 genes FDR < 0.1) for the interaction term of treatment with time in vitro. Genes are shown at baseline (control, left) and after rapamycin treatment (right). (f, g) Effect of Rapamycin on aging pathways. Gray bars represent slope of linear regression with time in vitro (in years, vst‐normalized). Yellow bars represent treatment slope. A yellow slope of 0 indicates that rapamycin fully (100%) rescues the aging pathway, and is no longer up‐ or downregulated. For each pathway, gene‐wise baseline slopes and treatment interaction slopes were extracted, and compared using paired *t*‐test. *p*‐values were adjusted for multiple testing. Average slopes per pathway were plotted as a bar graph (Repeated measures ANOVA followed by Dunnet's multiple comparisons test; downregulated: *F*(1.9, 30) = 108.2, *p* < 0.0001, Control vs. Metformin *p* < 0.0001, Control vs. Rapamycin *p* < 0.0001; upregulated *F*(1.3, 7.4) = 2.4, *p* = 0.16, Control vs. Metformin *p* = 0.01, Control vs. Rapamycin *p* = 0.7). (h) Percent rescue defined as (−Mean baseline slope/Mean Interaction slope) * 100. (i) Progression of average gene expression of each individual aging pathway at baseline, and after Metformin and Rapamycin treatment. Simple linear regression.

## Data Availability

The bulk RNA‐seq, single‐cell RNA‐seq, and DNAm raw data generated in this paper are accessible through ArrayExpress E‐MTAB‐16089, E‐MTAB‐15859, E‐MTAB‐15924, and E‐MTAB‐15993 (https://www.ebi.ac.uk/biostudies/arrayexpress). Any additional information required to reanalyze the data reported in this paper is available from the lead contact upon request.
