# Supplementary material for: Long‐Term Post‐Mitotic Culture of Human Fibroblasts Recapitulates and Accelerates Features of In Vivo Aging and Enables Dynamic Testing of Anti‐Aging Compounds
Source: Aging Cell. 2026 Nov 1;25(11):e70796. doi: 10.1111/acel.70796 (PMC13724222; doi:10.1111/acel.70796)
Supplement: Supplementary file 1 [file ACEL-25-e70796-s004.docx]

**Long-Term Post-Mitotic Culture of Human Fibroblasts Recapitulates and Accelerates Features of In Vivo Aging and Enables Dynamic Testing of Anti-Aging Compounds**

Larissa Traxler^1,2,3#^, Lukas Karbacher^,1,2,3#^, Oliver Borgogno^1,2,3^, Taylor Ozbun^1^, Kylie Champion^1^, Anna Smaling^4^, Lucia Zhou-Yang^1,2,3^, Joseph R. Herdy^3^, Lena Böhnke^4^, Barbara C. Böckle^5^, Fred H. Gage^3^, Hildegard I.D. Mack^6^, Michaela Defrancesco^7^, Jerome Mertens^1,2,3,4*^

^1^ Department of Neurosciences, University of California San Diego, La Jolla, CA, USA.

^2^ Sanford Consortium for Regenerative Medicine, University of California San Diego, La Jolla, CA, USA.

^3^ Laboratory of Genetics, The Salk Institute for Biological Studies, La Jolla, CA, USA.

^4^ Institute for Molecular Biology, University of Innsbruck, Austria

^5^ Department of Dermatology, Venereology and Allergology, Medical University of Innsbruck, Innsbruck, Tyrol, Austria

^6^ Department of Biochemistry and Genetics of Aging, Research Institute for Biomedical Aging Research, University of Innsbruck, Innsbruck, Tyrol, Austria

^7^ Division of Psychiatry I, Department of Psychiatry and Psychotherapy, Medical University of Innsbruck, Innsbruck, Tyrol, Austria.

\# equal contribution

*Correspondence to: [jmertens@health.ucsd.edu](mailto:jmertens@health.ucsd.edu)

**Supplementary Figure 1. Aging clocks in-vitro and in-vivo.** (a) Overview of fibroblasts used in in-vivo aging. (b) Overview of starting fibroblasts for in-vitro aging. (c) Immunofluorescence staining (p16) of fibroblasts at Month 0 and 6. Positive control represent senescent fibroblasts (Etoposide, 20 µM, 48hours). (d) Quantification of nuclear Area in fibroblasts undergoing replicative senescence (passage 30+), and at Month 0, 3, and 6 of non-replicative in-vitro aging. One-way ANOVA (F(3,10)=11.3, p=0.0015). (e) Gene expression of major SASP factors (scaled per row, vst normalized) in an independent dataset of proliferative and senescent fibroblasts (GSE63577, Marthandan et al. 2016) and non-replicative in-vitro aging (Month 0 – 6). Average expression (vst-normalized) of all SASP factors per donor and timepoint are represented as dots above heatmap. (f) DNAm age was calculated using the Online New Methylation age Calculator for the in-vivo cohort of donors between 0 and 92 years of age (n=22). Pearson correlation of chronological age and estimated age calculated using Blood&Skin DNAm age, Hannum clock and PhenoAge calculator. (g) Clock accuracy defined as the difference between estimated age and chronological age for DNAm clocks. Repeated measures ANOVA (F(1.6, 24.0)=50.45, p<0.0001). (h-k) Calculation of transcriptomic age in our small in-vivo dataset (n=20, Supplementary Fig.1a) and the Fleischer et al. aging dataset. Pearson correlation was calculated on each dataset individually and after merging both datasets. (h) RNAAgeCalc - across-tissue (i) RNAAgeCalc - fibroblast-specific (j) tAge and (k) the transcriptomic age of Peters et al. 2015. (l) Relative transcriptomic aging clock on merged in-vivo and Fleischer et al. datasets comparing donors ≤ 26 years or ≥68 years (n=50 per group), and on the in-vitro aging dataset comparing baseline (M0) and six months of in-vitro aging (M6). (m) Linear mixed effect model (DNAm ~ Age ~ (1|Donor)) of Blood&Skin DNAm Age. Simple Linear Regression was performed to assess slope. (n) DNAm age at baseline (M0) and after six months of in-vitro aging (M6). Each dot represents an independent donor. Paired t-test (t(3)=3.3, 95%CI [0.22,10.7]). (o) RNAAgeCalc - across-tissue of the in-vitro aging transcriptomic dataset (n=4 independent donors). Blue line represents slope of same transcriptomic clock based on in-vivo aging (small cohort). Linear mixed effects model on RNAAgeCalc - across-tissue. Residual age was calculated by subtracting the chronological age from the RNAAge. (p) RNAAgeCalc - across-tissue at baseline and at Month 5 of in-vitro aging. Paired t-test (t(3)=4.375, 95%CI [5.9, 37.2]). tAge at baseline and at Month 6 of in-vitro aging. Paired t-test (t(3)=2.6, 95%CI [-6.5, -59.8]). Each dot represents an independent donor.

**Supplementary Figure 2. Transcriptomic changes during in-vitro aging.** (a) Linear regression of in-vitro aging (adjusted p<0.05), in-vivo aging (p<0.05) and Fleischer et al. (adjusted p<0.05) across age/time in-vitro. Overlap of significant genes. (b) Up- and downregulated in-vitro aging genes were ranked by slope (logFC, limma) and tested for enrichment in in-vivo datasets, and vice versa. Similarly, in-vivo aging gene rankings were tested across independent in-vivo datasets, demonstrating strong reciprocal enrichment. (c,d) Significant genes from all three datasets were included in a shared pathway analysis using ClueGO with a common ontology-based network and identical statistical parameters. Pathways with significant term p-values are shown; dot size represents the percentage contribution of the aging gene set to each pathway. All identified pathways were further tested for enrichment using fgsea (R), and the dot color corresponds to the –log_10_(p-value) from the fgsea analysis. (e,f) Vst-normalized expression was averaged per pathway (overlapping pathways from Fig.2b), and linear regression was used to estimate pathway-level slopes (vst-normalized counts). Statistical comparison of slopes was performed in R. To assess pathway-level differences between in-vitro and in-vivo aging at gene-level, we fit a linear model including an interaction term between age and dataset (Age x Dataset) with in-vitro aging as reference. Interaction p-value was corrected for multiple testing using the Benjamini-Hochberg method. (g) Linear regression slope across in-vitro aging (green), in-vivo aging (blue), or Fleischer in-vivo aging (brown) of three representative accelerated KEGG pathways in-vitro, each dot representing the slope of a gene contributing to this pathway. (h) Box plot of slopes (vst-normalized counts, years) across all genes in the dataset. (i) GSE113957 divided into young (<40 years, n=67) and old (>40, n=76). Pearson correlation of slopes (vst-normalized, years) after removal of 1.5 IQR outliers (linear regression, adjusted for sex). (j) Fibroblasts with inducible expression of Ascl1 and Ngn2 were maintained in non-proliferative culture for up to 6 months before conversion induction (doxycycline). Transcriptomic age was assessed at day 21 after conversion induction (n=3 independent donors) in iNs at baseline (M0) and after 6 months of in-vitro aging (M6). RNAAgeCalc - across-tissue. Years aged was calculated by subtracting RNAAge at baseline from month 6. Unpaired t-test (t(9)=3.2, 95%CI [2,12]). (k) DNAm (n=3 at M0, n=2 at M6) and transcriptomic age clocks (n=3 per group) were assessed in iNs generated from replated fibroblasts. Fibroblasts were replated at baseline (M0) or after six months of pre-aging (M6) before direct conversion to iNs. Paired t-test. Each dot represents an individual donor. Lines connect donors at different timepoints. (l-m) Years aged in iNs and fibroblasts from the same donors (n=3) as calculated by subtraction of estimated age at baseline from age at month 6 (month 5 in RNAAgeCalc - across-tissue). Paired t-test. Average estimated age per group is indicated in the graph.

**Supplementary Figure 3. Single cell RNA sequencing of in-vitro aging.** (a,b) Cell number and reads per cell for each donor (n=2) at Month 0 and Month 5. (c) Proportion of cells contributing to each cluster, showing a clear annotation of each donor at each time-point. Each donor shows a minimal and comparable number of cells in G2M phase. (d) RNA age calculator age prediction for scRNA-seq micropools (50 cells per micropool) analyzed using Seurat. Ordinary one-way ANOVA and Sidak’s multiple comparison test (F(3,537)=225, p<0.0001, #4 M0 vs M5 p<0.0001, #3 M0 vs M5 p<0.0001). (e-g) Aging score calculated using Seurats AddModuleScore, based on genes extracted from in-vitro aging linear regression (adjusted p<0.05), the RNA age calculator – no tissue, and Fleischer et al. linear regression (adjusted p<0.05) (Supplementary Table 2). Cells in the lower 50 % quartiles were considered young (purple), and cells contributing to the upper 50 % were considered old (green). Pie charts show a quantification of the number of cells contributing to each quartile. (h) Negative binomial deviance representing cell-to-cell heterogeneity of aging scores. Wilcoxon rank-sum test revealed a significant difference in NB deviance between M0 and M5 (p<0.0001). Wilcoxon rank-sum test comparing Δslope (M5 − M0) between aging pathway genes and background genes showed a significant enrichment in aging pathway genes (p<0.0001).

**Supplementary Figure 4.** **Anti-aging drug Rapamyn showing limited effects on human cell aging** (a) DNAm Blood&Skin clock in Control (grey), Metformin-treated (red) and Rapamycin-treated (yellow) cultures. Simple linear regression, comparing slopes. Statistical comparison was performed using a mixed linear effect model (treatment x time in-vitro: estimate =-0.18 ± 0.09, t(49)=-2.1). (b) Aging velocity based on DNAm age per donor (in years; Paired t-test: t(3)=1, 95%CI [-30, 16], p=0.39). Years aged in-vitro calculated by subtracting the age of Month 0 from Month 6 in Control (grey) and Rapamycin-treated cultures (yellow). (Paired t-test: t(3)=1.0, 95%CI [-10, 5], p=0.38). (c) RNAAgeCalc - across-tissue on Control (grey), Metformin- (red) and Rapamycin- (yellow) treated cultures. Simple linear regression was performed to determine difference of slopes. Violin plots of slopes (in years) and years aged in-vitro, repeated measures one-way ANOVA (F(2) = 6.7, p=0.06) and Sidak’s multiple comparisons test (Control vs Metformin p=0.055, Control vs Rapamycin p=0.37). (d) Effect of anti-aging treatment on tAge. Repeated measures ANOVA with Dunnet’s multiple comparisons test. Velocity: F(1.1, 3.3)=0.04, p=0.86. Years aged: F(1.4, 4.11)=6.0, p=0.06). (e) Mixed-effects linear model was performed. The heatmap represents top 100 genes (sorted by smallest FDR, including 9 genes FDR<0.1) for the interaction term of treatment with time in-vitro. Genes are shown at baseline (control, left) and after rapamycin treatment (right). (f,g) Effect of Rapamycin on aging pathways. Grey bars represent slope of linear regression with time in-vitro (in years, vst-normalized). Yellow bars represent treatment slope. A yellow slope of 0 indicates that rapamycin fully (100%) rescues the aging pathway, and is no longer up- or downregulated. For each pathway, gene-wise baseline slopes and treatment interaction slopes were extracted, and compared using paired t-test. P-values were adjusted for multiple testing. Average slopes per pathway were plotted as a bar graph (Repeated measures ANOVA followed by Dunnet’s multiple comparisons test; downregulated: F(1.9, 30)=108.2, p<0.0001, Control vs. Metformin p<0.0001, Control vs Rapamycin p<0.0001; upregulated F(1.3, 7.4)=2.4, p=0.16, Control vs. Metformin p=0.01, Control vs Rapamycin p=0.7). (h) Percent rescue defined as (-Mean baseline slope/Mean Interaction slope) *100. (i) Progression of average gene expression of each individual aging pathway at baseline, and after Metformin and Rapamycin treatment. Simple linear regression.
